# Spatial variability of sedimentary assemblages reflects variations in bioerosion pressure of adjacent coral reefs

**DOI:** 10.1371/journal.pone.0311344

**Published:** 2024-10-11

**Authors:** Victor Rodriguez-Ruano, Richard B. Aronson, Lorenzo Alvarez-Filip, Esmeralda Perez-Cervantes, Nuria Estrada-Saldivar, William F. Precht

**Affiliations:** 1 Department of Ocean Engineering and Marine Sciences, Florida Institute of Technology, Melbourne, Florida, United States of America; 2 Biodiversity and Reef Conservation Laboratory, Unidad Académica de Sistemas Arrecifales, Instituto de Ciencias del Mar y Limnología, Universidad Nacional Autónoma de México, Puerto Morelos, México; 3 Bio-Tech Consulting Inc., Coastal and Marine Sciences, Miami Lakes, Florida, United States of America; National Cheng Kung University, TAIWAN

## Abstract

The composition of coral-reef sediments is highly variable across space and time, and differences in the life histories of the dominant calcifying organisms on reefs contribute to the heterogeneity of reef sediments. Previous studies have suggested that variations in coral-reef bioerosion can influence spatial and temporal variations of sedimentary assemblages: elevated erosion rates of dead coral skeletons can trigger a pulse of coral-derived sediments and cause a shift in the dominance of sedimentary grains from coralline algae, such as *Halimeda*, to coral. We assessed the variability of the sedimentary composition and bioerosion rates of reefs at different spatial scales to determine the association between these two variables. We surveyed the benthic assemblages on reefs exhibiting different ecological states and collected samples of the associated sediments. We calculated the carbonate budget for each site and compared their variability at different hierarchical levels to the variability of their respective sedimentary assemblages. At the scale of sites (1–10 km), *Halimeda* cover was a significant predictor of the relative abundance of *Halimeda* grains. Both the relative abundance of coral grains and reef bioerosion rates varied significantly at the scale of locality (tens to hundreds of km), with high abundances of coral grains in the sediments coinciding with high rates of bioerosion. The main drivers of bioerosion at our localities were parrotfish assemblages dominated by large size classes of excavating species such as *Sparisoma viride*. Reef sediments may reflect the gross degree of bioerosion pressure that reefs experience, and historical changes in bioerosion rates could potentially be assessed by examining the sediments across temporal scales.

## Introduction

Rates of coral-reef accretion are influenced by environmental variables such as water temperature, light availability, water chemistry, turbulence, and sedimentary inputs from adjacent terrestrial systems [1–3]. In addition, hurricanes, large-scale climatic events such as El Niños, and coral diseases can alter reef growth [4–6]. These factors can reduce coral growth rates and even decimate coral populations, tipping reefs into a net erosional state [7]. Using the fossil record, geologists have studied the growth-history of modern and relict reefs to understand how the magnitude and periodicity of these factors influence the ability of reefs to keep pace with sea-level rise [1, 8–10].

The fossil record of Holocene reefs is composed of preserved coral skeletons and, to a lesser extent, the skeletal remains of other calcifying taxa such as coralline algae, foraminiferans, mollusks, and echinoderms [11]. These skeletal elements are either cemented into the reef framework or bound together into loose carbonate aggregates (i.e., intraclasts) by crustose coralline algae and submarine cementation, or preserved in pockets of loose sediment [12, 13]. Because coral skeletons are the primary contributors to the reef framework, geological and palaeoecological studies typically use them as their primary data source [6, 14–16]. Sediments, however, are also a major component of reef accretion, and the sedimentary record of reefs can provide insights into their geology and ecology [17]. Sedimentary petrography, the classification of sediments based on their biological or geological origin, has been widely used to study geo-ecological processes of coral reefs [18]. For instance, Li et al. [19, 20] determined the hydrodynamics that control sedimentary transport between reef habitats by studying the sedimentary assemblages of reefs before and after storm events. Other studies have used sediment petrography to estimate the contribution of sediments of different origins to beach sand and island maintenance [21–24].

Furthermore, sedimentary petrography has been used to characterize population dynamics of corals and echinoderms within the fossil record and assess their historical influence on reef growth [25]. Greenstein [26] and Walbran et al. [27] studied the skeletal remains of the crown-of-thorns seastar *Acanthaster solaris* (formerly *Acanthaster planci*) in the Pacific and of the echinoid *Diadema antillarum* in the Caribbean in an attempt to identify previous mass-mortality events within reef sediments. Neither of them, however, provided conclusive results due to rapid taphonomic degradation, downward mixing of skeletal ossicles by bioturbation, and sedimentary overturning by cyclones [28–30].

Nevertheless, the coral and algal fractions of reef sediments have shown promising preservation potential within the sedimentary record. For instance, Precht and Aronson [31] surveyed the sedimentary composition and ecological state of a reef in Jamaica for 20 years. They observed a shift in dominance from the coralline green alga *Halimeda* to coral-derived grains, which coincided with a decrease in coral cover on the reef from 75% to 5%. Similarly, Lidz and Hallock [32] collected sediment samples across the Florida reef tract. They reported that sediments adjacent to reefs that had experienced high coral mortality were dominated by coral grains, whereas sediments adjacent to reefs that had retained high coral cover were dominated by *Halimeda* grains. These studies suggest that living corals sequester large amounts of CaCO_3_ to build their skeletons, which remain “locked up in long-term growth” [31]. By contrast, other calcifying organisms, such as coralline algae, exhibit short lifespans and high population-turnover rates, which promote high fluxes of coralline-algae fragments that become the dominant constituents of reef sediments [33–35]. When stressors cause extensive coral mortality, however, the dead coral skeletons become available to bioeroders, and bioerosion should increase the relative abundance of coral grains [7, 32].

Although Lidz and Hallock [32] hypothesized that the main process that drove the high abundance of coral grains on degraded reefs was the bioerosion of the dead framework, they did not have estimates of bioerosion to complement their sedimentary analysis. We sought to determine whether variability in the composition of reef-sediment assemblages across spatial scales could be attributed to gross levels of reef bioerosion. We sampled spur-and-groove reef habitats with varying levels of coral cover and bioerosion pressure in the northern Mexican Caribbean. At each site, we conducted ecological surveys on the spurs. These surveys assessed percent benthic cover of calcifying taxa, as well as the abundance of bioeroders such as sea urchins, excavating sponges, and parrotfish. We used the surveys to estimate calcification and bioerosion rates and calculate the carbonate budget for each site. Additionally, we sampled the sediments from sediment-pockets at the tops of spurs and from the adjacent grooves at each site and analyzed them petrographically.

We hypothesized that the reef sediments would reflect reef-carbonate budgets in two ways. First, the spatial trends of sand-sized coral grains would reflect the trends exhibited by reef bioerosion rates, indicating that bioerosion is a major driver of the spatial variability of reef sediments. Second, the relative abundance of sand grains from other calcifying taxa, such as coralline algae, would exhibit a positive relationship with the benthic cover or abundance of their respective living populations, given the high population-turnover rates that these organisms exhibit. Understanding variations in reef-sediment assemblages and their relationship with reef geo-ecological processes should enable us to determine the utility of reef sediments as potential indicators of reef processes, such as bioerosion, as suggested by Lidz and Hallock [32].

## Materials and methods

### Study sites

We surveyed the spur-and-groove habitats of seven reefs at 4–12 m depth along the northern coast of the Mexican Caribbean. The spur-and-groove geomorphology consists of prominent buttresses of reef framework—the spurs—which run perpendicular to the coast and alternate with sand channels—the grooves. Our sites were nested within three major localities: Punta Maroma, Akumal, and Punta Allen (S1 Fig). In the northernmost locality, Punta Maroma, we surveyed the spur-and-groove habitat of the site Mar F5, which had an average depth of 4 m. In Akumal, we surveyed three sites: Yal Ku (12 m), Dicks (8 m), and Langosta (10 m). In Punta Allen, the southernmost locality, we surveyed three additional sites: Punta Allen Norte (10 m), Punta Allen Centro (11 m), and San Antonio (6 m). Punta Allen is located within the Sian Ka’an Biosphere Reserve, and our surveys were conducted under permit F00.9.DRBSK/094/2021, which was issued by Mexico’s National Commission of Natural Protected Areas (CONANP). All sites were surveyed during the spring and summer months (March–June) of 2021.

The oldest ecological surveys at the three localities date back to 1986 [36]. Absolute coral cover in fore-reef habitats 5–10 m deep, which corresponds to the spur-and-groove zone, ranged from 19% at Punta Maroma to 28% at Punta Allen 35 years ago. Although the descriptions of these surveys did not specify whether they were performed on a spur-and-groove habitat, the depth at which the surveys were conducted on the fore-reef suggests that it is highly likely that the surveys account for the spur-and-groove assemblages of these sites. The dominant coral taxon at Punta Maroma and Akumal was the branching coral *Acropora palmata* (average absolute *Ac*. *palmata* cover: 11.2% and 9.2%, respectively), and the dominant taxon at Punta Allen was the foliose genus *Agaricia* spp. (9.2% absolute cover). At Punta Maroma, the second-most abundant taxon was *Agaricia* spp. (4.2%), followed by massive corals in the family Mussidae (2.1%) and *Orbicella* spp. (1.5%). At Akumal, the second-most abundant taxon was *Ag*. *tenuifolia* (5%), followed by *Ac*. *cervicornis* (4.2%) and massive corals in the Mussidae family (2.1%). At Punta Allen, *Ac*. *palmata* was the second-most abundant coral taxon (6.4%), followed by *Ag*. *tenuifolia* (4.8%) and *Orbicella* spp. (3.6%).

### Ecological surveys

To survey the contemporary benthic assemblage at each site, six 10-m transects were deployed using fiberglass surveyor’s tapes stretched over the bottom. Each transect was placed in the center of an individual spur along its main axis. Transects were placed at least two spurs (approximately 30 m) apart to avoid overlap in sampling the sediments (see below). Following the Reef Budget methodology developed by Perry and Lange [37], we used the line-intercept method to quantify the percent cover of each benthic component. A second, flexible measuring tape was conformed to the three-dimensional surface profile of the reef underlying the horizontally stretched, 10-m transect tape. The three-dimensional length of each benthic component (in cm) was then measured across all linear 10 meters of the stretched transect tape. To estimate percent cover for each transect, we calculated the proportional 3-dimentional length covered by each benthic category relative to the final, total length of the 3-dimentional profile of the reef across the 10-m linear transect. The ratio of the total length of the 3-dimentional profile of each transect to the total length of the 10-m transect was used to calculate the reef’s rugosity. We identified corals to the genus- or species-level when possible and coralline algae as erect/articulated, *Halimeda* spp., or crustose coralline algae (CCA). For the remaining benthic components, we used more general, higher-level identifiers such as sponges, soft corals, fleshy macroalgae, turf algae, rubble, dead coral, and sand.

We also surveyed excavating sponges and sea urchins within a total area of 10 m^2^ along each benthic transect by recording occurrences within 0.5 m on either side of each transect tape. We identified each sea urchin and measured its test diameter using calipers, and we estimated the surface area covered by excavating sponges by measuring their maximum lengths and widths visible at the surface of the substrate. Additionally, we deployed eight 30 x 2-m transects perpendicular to the shoreline to survey parrotfish assemblages. These transects were set along the main axes of the spurs that were flanking the spurs where we deployed benthic transects. We swam at a slow, steady pace along each transect, recording parrotfish within a meter of each side of the transect. We identified each individual that swam within the transect area to the species level and visually estimated its total length (cm) and life-stage (i.e., juvenile, intermediate phase, or terminal phase).

We used our ecological data to calculate the carbonate budget for each site using the Reef Budget V2 methodology for Caribbean reefs [37]. This carbonate-budget model uses published values for skeletal density (g cm^-3^) and linear extension rates (mm yr^-1^) to estimate the calcification rates of each coral species. These calcification rates also take into consideration the average colony morphology of each coral species, because many coral species, especially those with branching morphologies, actively calcify at their branch tips but exhibit lower calcification rates across the rest of the colony [38].

For sea-urchin bioerosion rates, Reef Budget uses equations derived from the relationship between test size and sediment production for each sea-urchin species. Bioerosion rates are derived from previous studies that have looked at the amount of CaCO_3_ present in sea-urchin gut contents and fecal pellets [37, 39–41]. For sponge bioerosion rates, Reef Budget uses the relationship between sponge surface-area (cm^2^) and the rates of physical and chemical erosion (mg CaCO_3_ cm^-2^ d^-1^) calculated by de Bakker et al. [42]. Parrotfish bioerosion is calculated by using previously published, species-specific, size-specific, and life-stage-specific bite rates and estimates of substrate removal for scraping and excavating species [37, 43–45]. Since parrotfish are highly mobile, their bioerosion pressure is assumed to be reef-wide. Therefore, the average, reef-wide estimate of parrotfish bioerosion is estimated and applied to each benthic transect. Microbioerosion is calculated by applying the average rate that was calculated for the Caribbean region to the three-dimensional surface-profile of the substrate that is affected by microbioerosion (i.e., dead coral, rubble, turf, cyanobacteria, and the substrate below coralline algae).

### Sedimentary surveys

We collected nine sediment samples along each benthic transect by scooping them with 100 mL plastic jars. Up to three of these samples were collected from sedimentary pockets found on top of the reef spur being surveyed, at intervals of approximately 5 m. In addition, three samples were collected from each of the two grooves flanking the spur, totaling six groove-sediment samples per transect. To remove all organics, we rinsed the sediment samples in fresh water, soaked them in a 5% sodium hypochlorite solution for 24 hours, and oven-dried them at 80 ºC for 12 hours. The sediment samples were then impregnated in blue epoxy resin, cut into thin sections (30 μm), and mounted onto slides for analysis under a petrographic microscope. We used a randomized point-count method to sample 200 sand grains per slide to assess the relative abundance of the various sedimentary components based on their biological or geological origins.

### Data analysis

Before analyzing the data, we compared the sedimentary assemblages between samples collected on spurs and samples collected in the grooves to determine whether reef habitat was a factor that should be taken into consideration. We ran a permutational analysis of variance (PERMANOVA) with the Bray-Curtis similarity index and 9999 permutations to determine the difference in sedimentary assemblages based on the reef habitat (i.e, spur or groove). This preliminary analysis determined that there were no significant differences between samples collected on spurs and in the adjacent grooves (permuted F_1,16_ = 0.27, R^2^ = 0.02, p = 0.96); therefore, we ran our main analyses using the sediment samples collected from the grooves. We chose to analyze the samples from the grooves because in many instances there were not enough sediment-pockets found along the spurs of the transects to collect three samples per transect. In contrast, we successfully collected sediment samples from all the grooves flanking all of our transects.

We ran a nested PERMANOVA with the Bray-Curtis similarity index and 9999 permutations to determine the difference in sedimentary assemblages among localities and sites nested within the localities. The dependent variable for this analysis was the relative abundance of each sedimentary component. Because the data were proportional, an arcsine square-root transformation was used to minimize the influence of extreme values. We then used a similarity percentages (SIMPER) test to identify the main variables that were driving the differences among localities and sites; to account for multiple comparisons, we ran 9999 permutations to generate a null distribution to compare with the test statistics of our data. We also used a non-metric multidimensional scaling (nMDS) plot to visualize the degree of separation among groups and determine the main sedimentary components that could be driving the separation among sites.

We used linear mixed-effects models (LMMs) to assess the relationship between coral sand grains (predictor variable), coral cover, and carbonate production (response variables). To assess the relationship between coral sand grains (predictor variable) and bioerosion (response variable) we used a generalized linear mixed-effect model (GLMM). We chose sand grains as the predictor variable because the goal of this model was to determine whether the abundance of coral sand grains can be used to predict states of bioerosion pressure on coral reefs. We treated site as a random factor and specified that it was nested within the locality variable. We initially ran the model using our raw, continuous data; however, because our bioerosion estimates exhibited a bimodal distribution (S2 Fig), we also ran a GLMM with our bioerosion data in binary form using a logistic GLMM with a logit-link function. Any bioerosion rate less than 1.0 kg CaCO_3_ m^-2^ yr^-1^ (mean = 0.53; range = 0.36–0.95) was set to 0 to represent low bioerosion pressure, whereas any erosion rate greater than 1.0 kg CaCO_3_ m^-2^ yr^-1^ (mean = 1.66; range = 1.04–2.27) was set to 1 to represent high bioerosion pressure. These benchmarks were established based on trends in bioerosion rates reported for the western Atlantic, Indian, and Pacific Oceans, which generally report bioerosion rates between 1.0 and 3.5 kg CaCO_3_ m^-2^ yr^-1^, with the sites that experience the lowest bioerosion rates having values below 1.0 kg CaCO_3_ m^-2^ yr^-1^ [46–48].

We also ran nested analyses of variance (ANOVA) that compared the variance among sites with the variance among localities for the rates of bioerosion, carbonate production, and the abundance of coral grains, with sites nested within localities. For this analysis, the bioerosion and carbonate production rates were log-transformed to conform to the assumptions of ANOVA of normality and homoscedasticity of ANOVA. The raw coral-grain abundances, on the other hand, met the ANOVA assumptions and there was no need to transform them. Since previous studies suggested that bioerosion was the main driver of upticks in coral sand grains [31, 32], the main objective of these ANOVAs was to identify the hierarchical level at which each variable exhibited the most variability. This analysis shed light on the factors that may be driving the spatial heterogeneity of coral-grain abundances at different scales [49, 50].

We chose the coralline green alga *Halimeda* to test the relationship between the living assemblage of non-coral, calcifying taxa and their sediments. *Halimeda* was abundant at our sites, both in the living and sedimentary assemblages. Unlike other taxa of coralline algae, it was easily identifiable to the genus-level in the sedimentary record. Furthermore, previous assessments of Caribbean sediments have determined that *Halimeda* was the dominant sedimentary component on Caribbean reefs before widespread degradation [31, 32, 51, 52]. We used a GLMM to assess the relationship between *Halimeda* sand grains (predictor variable), and live *Halimeda* cover (response variable), treating site as a random factor. All multivariate analyses and linear models were developed using the “vegan”, “BiodiversityR”, and “nlme” packages in R version 4.2.2 [53–56].

## Results

### Coral assemblages

Although there was considerable variability in coral cover among localities, there was no significant difference detected among localities due to high variability within sites at each locality (Locality: F_2,4_ = 6.21, p = 0.06; Site F_4,35_ = 1.62, p = 0.19; S1 Table). Mar F5, our site in Punta Maroma, was the site with the highest average coral cover (28.3 ± 3.5%, mean ± SE; S3 Fig), and San Antonio, a site in Punta Allen, was the site with the second-highest average coral cover (24.1 ± 3.0%). Langosta and Yal Ku, two sites from Akumal, were the ones with the lowest average coral cover (Langosta: 10.0 ± 1.7%; Yal Ku: 12.7 ± 2.5%; S3 Fig). The rest of the sites exhibited average coral-cover values between 18.4 and 18.9% (S3 Fig).

The coral assemblage at Mar F5 was dominated by *Porites* spp., and *Agaricia* spp., which exhibited average absolute coral covers of 18.0 ± 2.6% and 8.7 ± 2.8%, respectively. A similar trend was evident at the sites of Langosta (*Porites* spp.: 3.9 ± 0.9%; *Agaricia* spp.: 3.0 ± 1.0%), Yal Ku (*Porites* spp.: 2.2 ± 0.7%; *Agaricia* spp.: 7.2 ± 2.1%), and Punta Allen Centro (*Porites* spp.: 4.3 ± 1.8%; *Agaricia* spp.: 10.3 ± 3.3%). Although Punta Allen Norte was also dominated by *Porites* spp. (5.8 ± 1.6%) and *Agaricia* spp. (5.6 ± 1.1%), this site also had a considerable amount of *Orbicella* spp. (4.3 ± 1.9%). *Agaricia* spp. was the most prevalent coral taxon at the sites of Dicks and San Antonio (7.3 ± 2.2% and 11.1 ± 2.6, respectively); however, *Orbicella* cover was higher at these sites (Dicks = 6.8 ± 2.3%; San Antonio = 5.9 ± 5.6%) than *Porites* cover (Dicks: 3.2 ± 0.6%; San Antonio: 3.4 ± 1.3%).

### Carbonate budgets

There was no significant difference in gross carbonate production rates among localities or sites (Locality F_2,4_: 2.20, p = 0.23; Site F_4,35_ = 1.34, p = 0.27; S2 Table). By contrast, there was a significant difference in bioerosion rates among localities but not among sites (Locality F_2,4_: 95.88, p < 0.001; Site: F_4,35_ = 1.10, p = 0.37; Table 1). The bioerosion rates at Punta Maroma and Akumal exhibited similar values and were significantly higher than the bioerosion rates at Punta Allen (S3 Table; average bioerosion Punta Maroma: -1.61 ± 0.11 kg CaCO_3_ m^-2^ yr^-1^; Akumal: -1.68 ± 0.17 kg CaCO_3_ m^-2^ yr^-1^; Punta Allen: -0.53 ± 0.06 kg CaCO_3_ m^-2^ yr^-1^).

**Table 1:** Variation in bioerosion rates and coral grain abundance across spatial scales.

**Bioerosion**					
**Source**	**DF**	**Sum of Squares**	**Mean Square**	**F**	**p**
Locality	2	13.63	6.82	95.88	0.0004***
Site{Locality}	4	0.28	0.07	1.099	0.37
Error	35	2.27	0.07		
Total	41	16.18			
**Coral grains**					
**Source**	**DF**	**Sum of Squares**	**Mean Square**	**F**	**p**
Locality	2	606.6	303.29	14.77	0.01*
Site{Locality}	4	82.1	20.53	2.117	0.10
Error	35	339.4	9.70		
Total	41	1028.1			

Outputs for nested ANOVAs testing the difference in mean bioerosion rates and coral-grain abundance among localities and the sites nested within them. Significant results are highlighted in gray

The dominant bioeroder group varied among sites. Parrotfish were the dominant group at most sites, including Langosta (0.72 ± 0.41 kg CaCO_3_ m^-2^ yr^-1^), Yal Ku (1.45 ± 0.44 kg CaCO_3_ m^-2^ yr^-1^), Punta Allen Norte (0.21 ± 0.11 kg CaCO_3_ m^-2^ yr^-1^), and Punta Allen Centro (0.15 ± 0.09 kg CaCO_3_ m^-2^ yr^-1^). Excavating sponges were the dominant bioeroder group at two sites: San Antonio (0.33 ± 0.08 kg CaCO_3_ m^-2^ yr^-1^) and Mar F5 (0.53 ± 0.07 kg CaCO_3_ m^-2^ yr^-1^). Sea urchins were the dominant bioeroder group at Dicks (1.27 ± 0.21 kg CaCO_3_ m^-2^ yr^-1^).

**Fig 1:**
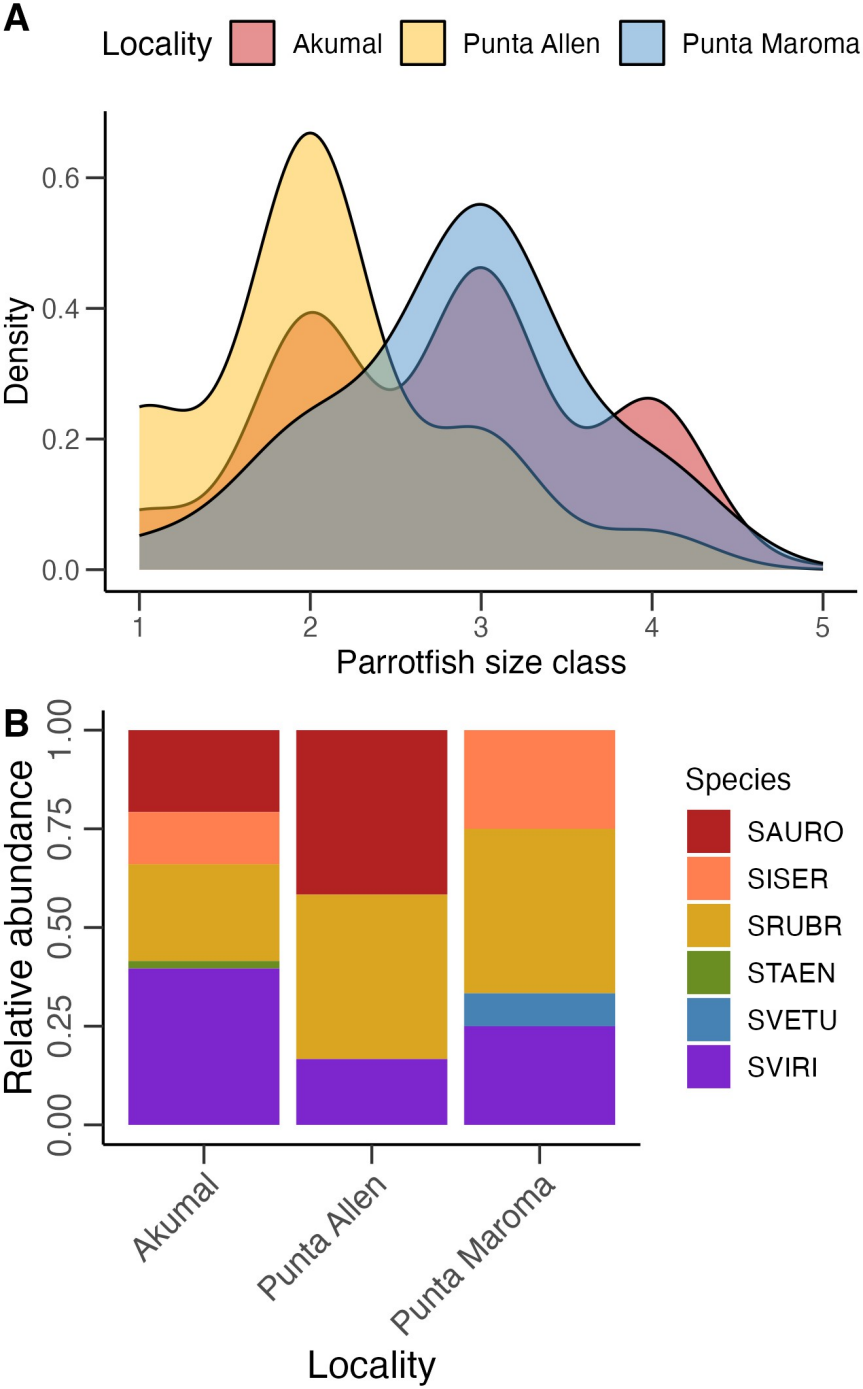
**(A) Size-class distributions of parrotfish assemblages (*Scarus* spp. and *Sparisoma* spp.) across localities.** Size class 1: 0–5 cm; size class 2: 6–10 cm; size class 3: 11–20 cm; size class 4: 21–30 cm; size class 5: 31–40 cm. Probability densities were calculated by dividing the frequency of each category by the total number of observations. **(B) Relative abundance for individuals of size class 4 (21–30 cm) for each species at each locality**. SAURO: *Sparisoma aurofrenatum*, SISER: *Scarus iseri*, SRUBR: *Sparisoma rubripinne*, STAEN: *Scarus taeniopterus*, SVETU: *Scarus vetula*, SVIRI: *Sparisoma viride*.

Although the sites at Punta Allen had similar parrotfish abundances to the other sites in Akumal and Punta Maroma (F_2,4_ = 0.09, p = 0.92), the parrotfish assemblage at Punta Allen was comprised of individuals that were significantly smaller than the ones in Akumal (Kolmogorov-Smirnov test D = 0.37, p < 0.0001; Fig 1) and Punta Maroma (Kolmogorov-Smirnov test D = 0.51, p < 0.0001; Fig 1). The parrotfish assemblages of Punta Allen exhibited a right-skewed distribution, with most individuals belonging to size class 2 (6–10 cm). By contrast, the parrotfish assemblages of Akumal and Punta Maroma exhibited a Gaussian-like distribution, with most individuals belonging to size class 3 (11–20 cm) followed by size classes 2 and 4 (21–30 cm; Fig 1).

**Fig 2:**
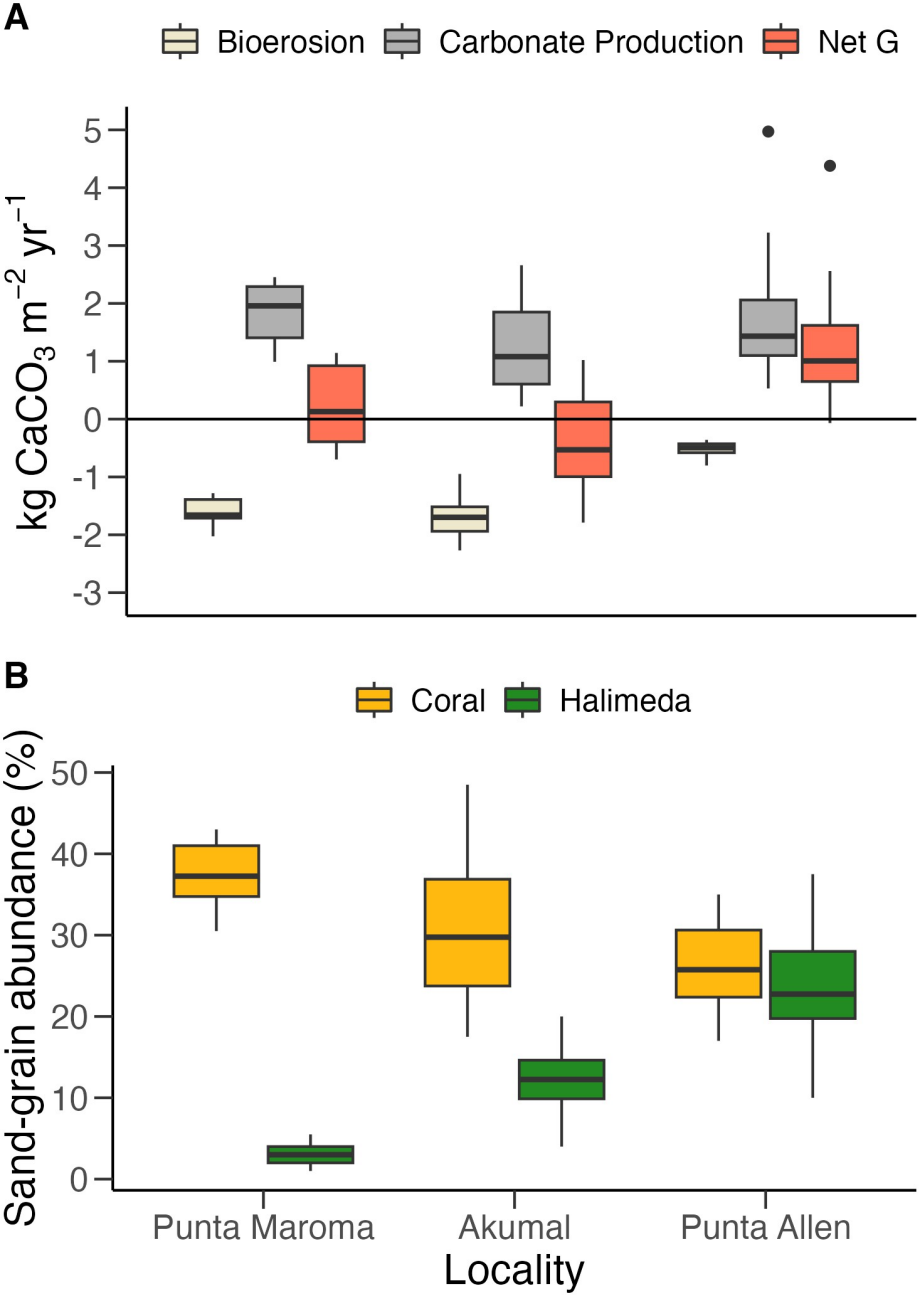
Comparison of carbonate budgets and sedimentary assemblages among localities. **(A)** Boxplot of the median (± interquartile range) of total bioerosion (tan), gross carbonate production (gray), and net carbonate production (red) for each locality. All rates are reported in kg CaCO_3_ m^-2^ yr^-1^. The black horizontal line delimits net production and net erosion. Black points represent statistical outliers. **(B)** Boxplot of median (± interquartile range) relative abundance of coral and *Halimeda* sand grains reported for sediments within each locality.

There was a significant difference in net carbonate-production rates among localities but not among sites (Locality: F_2,4_ = 18.91, p < 0.01; Site: F_4,35_ = 0.74, p = 0.57; S4 Table), which was likely driven by the variability in bioerosion rates among localities (Fig 2A). Net carbonate production was significantly higher at Punta Allen than at Akumal (Fig 2A; S5 Table). Two sites at Punta Allen exhibited the highest levels of *Orbicella* cover and the lowest bioerosion rates (Punta Allen Norte and San Antonio; S4 and S5 Figs); by contrast, two sites at Akumal had the lowest levels of net carbonate production and absolute coral cover, as well as the highest rates of bioerosion (Langosta and Yal Ku; S3 and S5 Figs). Based on the net carbonate-production rates, all the reefs within Punta Allen were experiencing net growth (S5 Fig), with Punta Allen Norte experiencing the highest rates of net carbonate production (1.5 ± 0.4 kg CaCO_3_ m^-2^ yr^-1^; S5 Fig). Punta Maroma was experiencing a slightly positive, but closer-to-neutral, rate of net carbonate production (0.2 ± 0.3 kg CaCO_3_ m^-2^ yr^-1^; Fig 2A). All three sites at Akumal exhibited net erosional states, with Yal Ku exhibiting the lowest rate of net carbonate production of all seven sites (-0.7 ± 0.3 kg CaCO_3_ m^-2^ yr^-1^: S5 Fig).

### Sedimentary assemblages

There were significant differences among sedimentary assemblages, with localities and sites accounting for 40% and 7% of the variability respectively (permuted F_2,4_ for locality = 28.91, R^2^ = 0.40, p = 0.0001; permuted F_4,77_ for site = 2.40, R^2^ = 0.07, p < 0.01; S6 Table). Mar F5 had the highest relative abundance of coral grains (37.4 ± 1.2%) and intraclasts (28.2 ± 0.8%), which, according to the SIMPER test, were the main variables that drove the separation of Mar F5’s sedimentary assemblages from the rest of the sites (S6 Fig; S7 Table). All three of the sites surveyed at Punta Allen had the highest relative abundances of *Halimeda* (Punta Allen Centro: 23.5 ± 1.3%; Punta Allen Norte: 20.9 ± 1.4%; San Antonio: 27.8 ± 2.0%; Fig 2B; S8 Table), which was the main driver separating them from Mar F5 (S6 Fig; S7 Table). Although *Halimeda* abundances at all the Akumal sites were lower than at the Punta Allen sites (Dicks: 11.8 ± 1.0; Langosta: 10.8 ± 1.1; Yal Ku: 14.5 ± 0.9; Fig 2B), only San Antonio was significantly different from Langosta and Dicks (S7 Table). All the Akumal sites were significantly different from one another; however, the differences among these sites were driven mostly by the variability of less-common grains such as octocoral sclerites, foraminiferal tests, and sea-urchin ossicles (S6 Fig; S7 Table).

**Fig 3:**
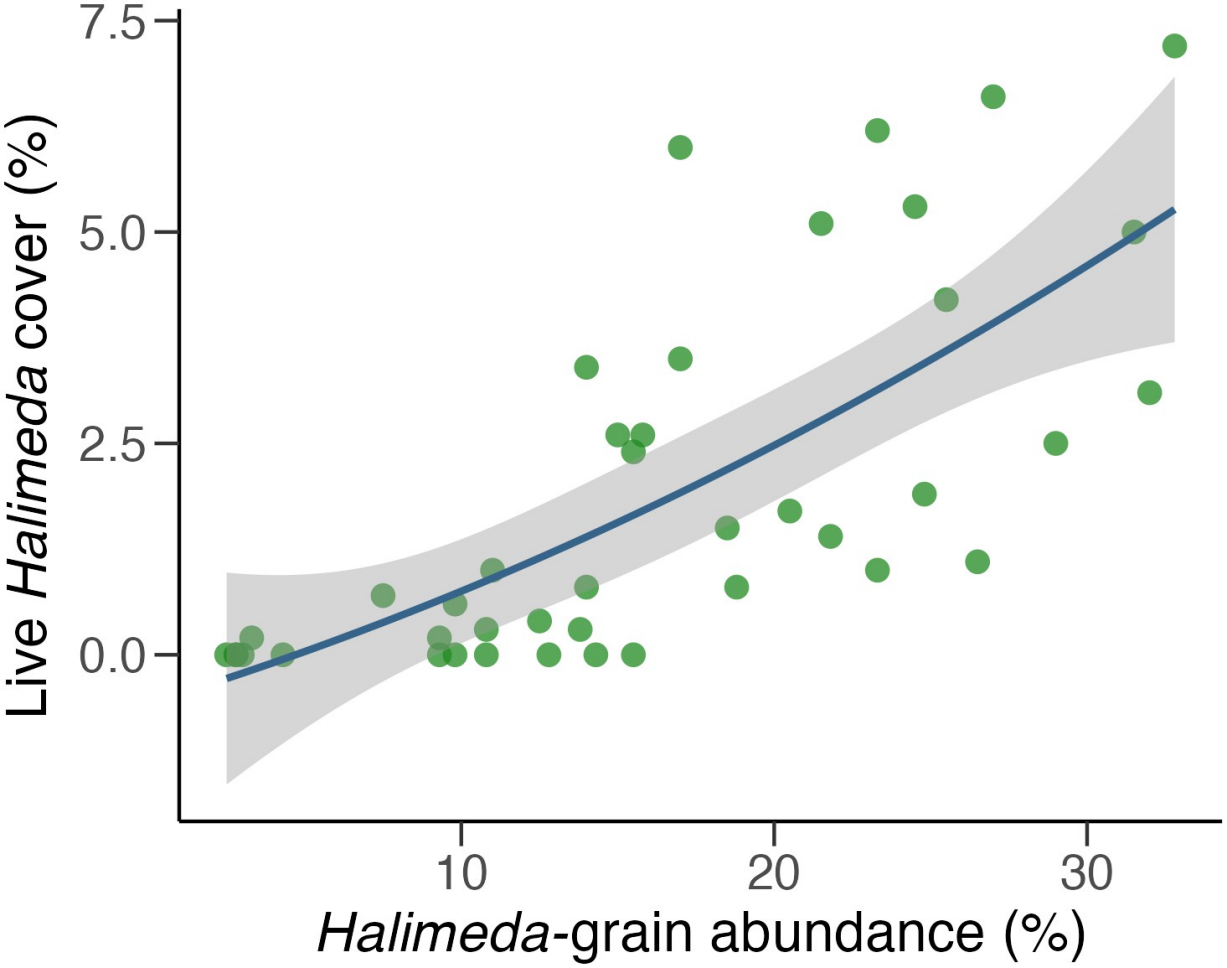
Relationship between the abundance of *Halimeda* sand grains and the percent benthic cover of live *Halimeda*. Scatterplot depicting the GLMM for the percentage of *Halimeda* grains in the sediments as a predictor of live *Halimeda* cover on the reef. The green dots represent the raw percent-cover values for *Halimeda* at each benthic transect (y-axis) and the relative abundance of *Halimeda* grains in the sediment samples as percentages (x-axis). The solid blue line represents the mean model fit, and the shaded area represents the 95% confidence interval of the fitted values.

According to the LMMs, the abundance of *Halimeda* grains was a significant predictor of the live *Halimeda* cover on the reef (fixed effect of *Halimeda* grain = 0.18, SE = 0.03, t_1,34_ = 6.40, p < 0.0001; Fig 3; S9 Table), yet the abundance of coral grains was not a significant predictor of coral cover nor carbonate production, (fixed effect of coral grain on coral cover = -0.18, SE = 0.35, t_1,34_ = -0.51, p = 0.61; fixed effect of coral grains on carbonate production = -0.04, SE = 0.03, t_1,34_ = -1.23, p = 0.23). Similarly, the logistic GLMM also determined that the abundance of coral grains was not a significant predictor of bioerosion (fixed effect of coral grain = -0.03, SE = 0.39, z_1,34_ = -0.08, p = 0.94). According to the nested ANOVA, however, there was a significant difference in the abundance of coral grains among localities but not among sites (Locality: F_2,4_ = 14.77, p < 0.05; Site: F_4,35_ = 2.12, p = 0.10; Table 1). These trends were similar to those observed for the bioerosion rates: Punta Maroma had the highest abundance of coral grains, followed by Akumal and Punta Allen (S10 Table).

## Discussion

The trends found across the different hierarchical levels revealed that both bioerosion rates and the abundance of coral grains varied significantly at the scale of locality (tens to hundreds of km; Table 1). Significant differences in bioerosion pressure were a major driver of the contrast between the positive growth of the reefs in Punta Allen and the neutral or eroding reefs in Akumal and Punta Maroma. The sites at Punta Allen exhibited the lowest rates of bioerosion, which can be attributed to high variability in the parrotfish size classes among localities. Indeed, parrotfish were a major contributor to bioerosion pressure at all sites and the variability of the dominant size-classes among localities coincided with the variability exhibited by rates of bioerosion and coral grains. These patterns suggest that variability in populations of bioeroding taxa, such as parrotfish, may be a major driver of overall bioerosion trends in the Mexican Caribbean and, therefore, a major control on the abundance of coral-derived sediments in the sedimentary record [57, 58].

The parrotfish assemblage of Punta Allen was dominated by small size classes (6–10 cm) that exerted low bioerosion pressure. In contrast, the parrotfish assemblages of Punta Maroma and Akumal were dominated by medium size classes (11–20 cm) that exerted high bioerosion pressure. These results agree with the trends described by Molina-Hernandez et al. [57] for parrotfish bioerosion in the Mexican Caribbean. They found that a recent increase in the net carbonate production of Mexican reefs was not necessarily driven by increases in coral cover, but rather by a decrease in bioerosion rates, especially from parrotfish. This decline in parrotfish bioerosion rates was driven by both a decline in parrotfish abundance and a shift in size-frequencies towards smaller individuals [57]. Our sites at Punta Allen had low carbonate-production rates [59, 60] but exhibited net-positive carbonate budgets because of the reduced bioerosion pressure exerted by small parrotfish. By contrast, our sites with similar or slightly lower carbonate-production rates in Punta Maroma and Akumal had net-neutral or net negative carbonate budgets because of the higher bioerosion pressure exerted by larger parrotfishes [45, 61].

Body size, however, is not the only factor that determines the intensity of parrotfish bioerosion. Species composition also greatly influences bioerosion intensity [43, 44, 61]. Parrotfish species are classified into four different types of grazers: browsers, excavators, scrapers, and croppers. Excavators and scrapers feed by foraging on short, productive algal turfs and CCA, and they can remove relatively large fractions of framework in the process; they, therefore, contribute the most to reef bioerosion [61]. Browsers and croppers, on the other hand, tend to feed on longer, filamentous turf algae and macroalgae; they do little-to-no damage to the reef framework, contributing the least to reef bioerosion [61, 62]. In the Caribbean, the predominant excavating species is *Sparisoma viride*, with previous studies reporting that large individuals can contribute to the majority of the bioerosion pressure recorded on reefs [57]. Our results support these previous findings: the parrotfish assemblage at Akumal not only had the highest abundance of large individuals, but also the highest proportion of large *S*. *viride* in comparison to Punta Allen and Punta Maroma. *Sp*. *viride* alone made up 40% of the large (21–30 cm) parrotfish assemblage at Akumal in comparison to 17% at Punta Allen and 25% at Punta Maroma (Fig 1B).

Other feeding behaviors that further influence the intensity of parrotfish bioerosion of reefs are the species-specific preferences for substrate on which they feed [61, 63]. For instance, *Sp*. *viride*, the main bioeroding species in the Caribbean, prefers to feed on convex surfaces. By contrast, *Scarus vetula*, another important bioeroder, prefers to feed on flat surfaces [61]. *Sp*. *viride* was a prevalent species at all localities while *Sc*. *vetula* was rare (Fig 1B). Although *Sp*. *viride* was more prevalent at Akumal, than Punta Maroma or Punta Allen, there were no significant differences in average reef rugosity among the localities we surveyed, with average rugosity values ranging from 1.39 at Akumal to 1.23 at Punta Maroma (Locality: F_2,4_ = 2.34, p = 0.21; Site: F_4,35_ = 1.78, p = 0.16). Therefore, rugosity is not a good estimate for assessing variations in parrotfish habitat/substrate preferences among our sites, at least with the methodology that we employed. Nevertheless, it is important to consider the influence of reef geomorphology on the dynamics of bioeroding taxa. Doing so would help develop more precise estimates of bioerosion pressure in different reef habitats, yielding high-resolution datasets and more accurate predictions of the response of reef dynamics to future disturbances.

The lack of baseline sedimentary data from previous years restricts our ability to estimate the temporal variability of sedimentary assemblages. Ecological assessments from 30–40 years ago, however, indicate that the fore-reefs in Punta Maroma, Akumal, and Punta Allen were dominated by *Ac*. *palmata* and *Ac*. *cervicornis* [36, 64], two historically important and abundant reef-building species on Caribbean fore-reefs [65–67]. The second-most important contributors to the reef-framework at these sites were massive corals of the genus *Orbicella* and brain corals of the family Merulinidae [36, 67, 68]. Disease outbreaks and thermal-stress events have caused a significant, Caribbean-wide decline in the abundance of these taxa over the last two decades [60, 64, 69–71]. Indeed, the presence of live acroporids at our sites was negligible and the abundance of *Orbicella* was also low (S4 Fig). Therefore, it is likely that as the dead skeletons became exposed to bioerosion, intense grazing pressure by parrotfish and the then-abundant sea-urchin *D*. *antillarum* (before its own decimation by disease in 1983–84 [72]) likely drove the pulse in coral-derived sediments in our sediment samples, similar to what Precht and Aronson [31] and Lidz and Hallock [32] observed.

High abundances of *D*. *mexicanum* in the eastern Pacific have been associated with drastic increases in bioerosion of the reef framework and subsequent sediment production [73–75]. During the 1982–83 El Niño event, high sea-surface temperatures caused a mass coral mortality event at Uva Reef, Pacific Panamá [76]. The dead coral skeletons were then overgrown by macroalgae, and this increase in food availability led to an outbreak of *D*. *mexicanum* [73]. The intense bioerosion that followed surpassed the rates of carbonate production of the reef and led to a significant loss of reef framework along with a pulse of coral-derived sediments [74, 75]. A more recent study by Perry et al. [58] used sediment-production rates from bioeroders to model sediment pulses caused by an increase in the abundance of parrotfish following a coral-bleaching event in the Maldives. They estimated that the increase in parrotfish biomass led to a three-fold increase in sediments produced by bioeroders [58]. Our study went further by using *in situ* ecological and sedimentological data to demonstrate that spatial variations in the abundance of coral-derived sediments reflected those of bioerosion rates at their respective localities.

Our bioerosion rates are estimates calculated at the site-level, derived from the relationship between the census data of macroborers and grazers and the average, published rates of carbonate removal recorded for those taxa [37]. The actual amount of carbonates removed from reefs, however, is highly variable due to the influence of other factors that are unaccounted for in our carbonate-budget model. Variations in the selectivity of substrate targeted by different bioeroders [63], the skeletal density and morphology of different coral species [77], and the accretionary and cementation activity of crustose-coralline algae (CCA) counteracting the erosion of the bare substrate are all factors that contribute to the spatial heterogeneity of framework erosion [78].

To overcome these limitations, a few studies have measured the true vertical erosion rates of different reef substrates through time. Molina-Hernandez et al. [78], for example, measured *in situ* vertical erosion rates on the dead skeletons of different coral species over two years. They determined that there was a significant difference in the amount of CaCO_3_ removed from skeletons of different species. *Orbicella*, a reef-building taxon, exhibited the highest erosion rates, but long-dead *Acropora* skeletons and calcareous hardgrounds did not change substantially. Furthermore, Molina-Hernandez et al. [78] suggested that CCA and thick, sediment-laden algal turfs sheeting the skeletons might provide some protection from grazers, causing a reduction in erosion rates. Kuffner et al. [79], on the other hand, measured *in situ* vertical erosion on *Orbicella* skeletons over 20 years and found high long-term erosion rates on the dead skeletons. Although CCA and thick algal turfs may have provided some protection and reduced erosion by grazers in the short term, persistent bioerosion pressure, especially from cryptic macroborers, was still acting upon the dead skeletons and causing a significant removal of the framework in the long term. In addition, mass coral mortality events opened space for bioeroders to exploit, and the early development of fine algal turfs on this open space, which are highly palatable to grazers, could have led to an increase in grazer abundance, promoting an increase in bioerosion pressure [73, 80].

Trends in total coral cover at our sites indicate that there has not been a significant change in Akumal and Punta Allen the last three decades. At Punta Maroma, however, coral cover has increased by 9% since 1986 [36]. Yet the presence of acroporids at our Punta Maroma site, Mar F5, is negligible, and it is now dominated by *P*. *astreoides* and *Agaricia* spp. The trends in coral-species composition paired with the high bioerosion rates at this site indicate that the coral grains encountered in our sediments may, therefore, reflect the erosion of the skeletons from acroporid and massive corals that perished over the last 30–40 years. Although Molina Hernandez et al. [78] determined that dead acroporid frameworks of Puerto Morelos experienced little to no vertical erosion in two years, they also argued that these patterns could be attributed to an increase in skeletal density from internal inorganic cementation, which may not correspond with higher erosion rates exhibited by recently dead skeletons. In addition, the branching skeletons of acroporids are vulnerable to fragmentation during storms [4]. Storms in previous years could have broken off a large number of dead fragments, which would have been deposited as rubble and eroded more easily than larger, denser, skeletal stumps that remained in growth position [66]. Thus, it is likely that the acroporid skeletons exhibited higher losses to erosion during the first years post-mortem in the late 1970s or early 1980s, producing the coral-grain signal we detected in the sedimentary record.

Trends in the composition of reef sediments described by Precht and Aronson [31] and Lidz and Hallock [32] suggest that reef sediments record degradation events on a scale of 20–30 years. Similar to the reefs in the Mexican Caribbean, the coral populations from the reefs they surveyed in Jamaica and Florida were decimated by disease, coral bleaching, and hurricanes [69, 81]. Those coral populations failed to recover to pre-disturbance conditions within the 20- to 30-year time frame of the studies, representing a long-term change in their ecological state. By contrast, other, more resilient reefs have suffered acute disturbance events, such as mass mortalities from coral bleaching or predator outbreaks but have recovered to pre-disturbance conditions within 7–13 years [82–84]. The variabilities in coral cover, resilience, and fish and invertebrate assemblages in coral reefs are driven by different processes across multiple spatial and temporal scales [41, 85, 86]. By sampling reef assemblages and the influential processes across multiple spatial and temporal scales, we can pinpoint the relative contributions of the major geological and ecological drivers of coral-reef heterogeneity.

Coral grains are not good predictors of carbonate production or coral cover, indicating that the coral sedimentary record does not reflect the living coral assemblage. In addition, the trends in spatial variability of carbonate production and coral cover are decoupled from the trends in the abundance of coral sand grains. There was no significant variability in coral cover and carbonate production among sites or localities (S1 and S2 Tables), unlike the correlated trends that bioerosion and coral sand grains exhibited (Table 1). It is important to note, however, that high rates of bioerosion do not necessarily connote a degraded reef. Highly productive reefs with high coral cover and high accretion rates can also exhibit high rates of bioerosion [46, 87]. Therefore, gross bioerosion rates derived from reef sediments cannot be used to discern the overall health of a reef.

Although reef sediments can vary significantly across reef habitats and sites, *Halimeda* grains seem to have been a dominant component of reef sediments at large scales throughout various Caribbean localities such as Jamaica [31], The Florida Keys [32], and Belize [21, 52] before the significant degradation of Caribbean reefs 50–70 years ago [88]. The reefs of Punta Allen conform to this pattern, but the abundance of coral grains tends to be slightly higher than the abundance of *Halimeda* grains (Fig 2B). Even though the reefs at Punta Allen experience low bioerosion rates and positive accretion rates, trends in benthic assemblages across the Mexican Caribbean suggest that they have lost a significant proportion of their reef-building corals and carbonate-production potential within the last four decades [36, 89]. Our recent surveys also indicate that the abundance and carbonate production rates of *Orbicella* spp. are much lower than those of other accreting Caribbean reefs [46, 59]. The high abundance of *Halimeda* coupled with the reduced dominance of coral grains in the sedimentary record suggests that even low levels of bioerosion can have a persistent, albeit weakened, influence on reefs that have failed to fully recover their coral assemblages to pre-disturbance levels.

Unlike the skeletal remains of *D*. *antillarum* and *A*. *solaris*, the abundance of *Halimeda* sand grains appears to be representative of the living *Halimeda* population [25]. Previous attempts to track acute episodes of *A*. *solaris* outbreaks and the demise of *D*. *antillarum* have detected pulses of their skeletal fragments in the surface sediments [26, 28–30]. As the skeletal fragments mix with time-averaged layers of sediment, however, this pulse dissipates as it is surpassed by the remains of the persistent, long-term contributors to the sedimentary record like *Halimeda* [25, 90]. Unlike echinoderms, *Halimeda* spp. are continuously growing and shedding old plates, depositing 3% of their total plates per day into the reef sediments and making major contributions to the sedimentary record [34, 35].

Our prospects for tracking population dynamics of calcifying taxa in the sedimentary record likely depend on their life histories and their prevalence in coral-reef ecosystems. Although sedimentary coral grains do not track changes in the living populations, they appear to reflect spatial trends in bioerosion pressure on reefs (Fig 2A and 2B). In addition to assessing the spatial variability in sedimentary assemblages, it is necessary to determine the temporal variability. Tracking changes in sedimentary assemblages through time in tandem with ecological data and records of influential disturbances would expand our understanding of residence times for autochthonous sediments and the degree of time-averaging that different reef habitats exhibit. Furthermore, understanding the spatio-temporal dynamics of sedimentary assemblages could be useful for tracking historical oscillations in bioerosion in the fossil record. Sediments from reef cores could then be used together with fossilized coral skeletons to paint a more detailed picture of the history of reef development.

## Supporting information

S1 Fig Map of the surveyed localities and sites within the Mexican Caribbean. The study sites within localities are shown as insets. These maps were generated using Natural Earth Data (http://www.naturalearthdata.com/), which were retrieved using the “rnaturalearth” package in R version 4.2.2.
(TIF)

S2 Fig Frequency distribution of bioerosion rates (in kg CaCO_3_ m^-2^ yr^-1^) recorded at all sites. (TIFF)

S3 Fig Average coral cover across sites. Bars represent the mean (± standard error) percent coral cover at each site we surveyed. Colors denote the locality for each site.
(TIFF)

S4 Fig Average benthic cover of weedy and framework-building taxa across sites. Bars represent the mean (± standard error) percent cover estimates for the dominant weedy (*Agaricia* and *Porites*) and framework-building (*Orbicella*) coral taxa at each site. Punta Allen N = Punta Allen Norte, Punta Allen C = Punta Allen Centro.
(TIFF)

S5 Fig Carbonate budget trends across sites. Boxplots represent the median (± interquartile range) total bioerosion (tan), gross carbonate production (gray), and net carbonate production (red) for each site. All of the rates are reported in kg CaCO_3_ m^-2^ yr^-1^. The black horizontal line delimits net production and net erosion. Black points represent statistical outliers.
(TIFF)

S6 Fig Variations in sedimentary assemblages among sites. The non-metric multidimensional scaling (nMDS) plot shows the relationship of sediment samples to the separation among sites. The species vectors shown are the sedimentary categories that contribute the most to the separation among sites. Squares represent the sites from Punta Maroma, circles represent the sites from Akumal, and triangles represent the sites form Punta Allen.
(TIFF)

S1 Table Variation in coral cover across spatial scales. Nested ANOVA results testing for differences in mean coral cover (%) among localities and the sites nested within them.
(DOCX)

S2 Table Variation in gross carbonate production across spatial scales. Nested ANOVA results testing for differences in mean gross carbonate production (kg CaCO_3_ m^-2^ yr^-1^) among localities and the sites nested within them.
(DOCX)

S3 Table Post-hoc, pairwise comparison of bioerosion rates across spatial scales. Tukey HSD post-hoc, pairwise comparisons of mean bioerosion rates of all localities. Significant comparisons are highlighted in gray.
(DOCX)

S4 Table Variation in net carbonate production across spatial scales. Nested ANOVA results testing for differences in mean net carbonate production (kg CaCO_3_ m^-2^ yr^-1^) among localities and the sites nested within them. Significant results are highlighted in gray.
(DOCX)

S5 Table Post-hoc, pairwise comparison of net carbonate production rates across spatial scales. Tukey HSD post-hoc, pairwise comparisons of mean net carbonate production rates of all localities. Significant comparisons are highlighted in gray.
(DOCX)

S6 Table Variation in sedimentary assemblages across spatial scales. Nested PERMANOVA results testing the degree of separation of sedimentary assemblages among localities and the sites nested within them. Significant results are highlighted in gray.
(DOCX)

S7 Table Post-hoc, pairwise comparison of sedimentary assemblages across spatial scales. Contrast tables of pairwise comparisons from the similarity of percentages (SIMPER) test on the abundance of sediment categories found for each site. The rows highlighted in gray indicate variables that were significantly different for the respective site pairs.
(DOCX)

S8 Table Summary of the composition of sedimentary assemblages across sites. Mean (± standard error) relative abundance of sediment grains for all the biological and geological categories found within 12 thin sections of sediments from each site.
(DOCX)

S9 Table *Halimeda* sediment grain abundance as a predictor of live *Halimeda* cover. Output from the linear mixed-effect model testing *Halimeda* grains in the sediment samples as a predictor of live *Halimeda* cover. Significant predictors are highlighted in gray.
(DOCX)

S10 Table Post-hoc, pairwise comparison of sedimentary assemblages across localities. Tukey HSD post-hoc, pairwise comparisons of the mean abundance of coral grains of all localities. Significant comparisons are highlighted in gray.
(DOCX)

## References

[1] Hallock P, Schlager W. Nutrient excess and the demise of coral reefs and carbonate platforms. Palaios. 1986;1: 389–398. doi: 10.2307/3514476

[2] Kleypas JA. Coral reef development under naturally turbid conditions: fringing reefs near Broad Sound, Australia. Coral Reefs. 1996;15: 153–167. doi: 10.1007/s003380050037

[3] Aronson RB, Hilbun NL, Bianchi TS, Filley TR, McKee BA. Land use, water quality, and the history of coral assemblages at Bocas del Toro, Panamá. Mar Ecol Prog Ser. 2014;504: 159–170. doi: 10.3354/meps10765

[4] Scoffin TP. The geological effects of hurricanes on coral reefs and the interpretation of storm deposits. Coral Reefs. 1993;12: 203–221. doi: 10.1007/BF00334480

[5] Rooney J, Fletcher C, Grossman E, Engels M, Field M. El Niño influence on Holocene reef accretion in Hawai’i. Pac Sci. 2004;58: 305–324. doi: 10.1353/psc.2004.0022

[6] Toth LT, Aronson RB, Vollmer S V., Hobbs JW, Urrego DH, Cheng H, et al. ENSO drove 2500-year collapse of Eastern Pacific coral reefs. Science. 2012;336: 81–84. doi: 10.1126/science.1221168 22767927

[7] Glynn PW. Bioerosion and Coral-Reef Growth: A Dynamic Balance. In: Birkeland C, editor. Life and Death of Coral Reefs. New York (USA): Chapman and Hall; 1997. pp. 68–95.

[8] Montaggioni LF. History of Indo-Pacific coral reef systems since the last glaciation: Development patterns and controlling factors. Earth Sci Rev. 2005;71: 1–75. doi: 10.1016/j.earscirev.2005.01.002

[9] Precht WF, Aronson RB. Stability of reef-coral assemblages in the Quaternary. In: Hubbard DK, Rogers CS, Lipps JH, Stanley GD, editors. Coral reefs at the crossroads. Dordrecht (Netherlands): Springer; 2016. pp. 155–173.

[10] Toth LT, Aronson RB. The 4.2 ka event, ENSO, and coral reef development. Clim Past. 2019;15: 105–119. doi: 10.5194/cp-15-105-2019

[11] Swinchatt JP. Significance of Constituent Composition, Texture, and Skeletal Breakdown in Some Recent Carbonate Sediments. SEPM J Sediment Res. 1965;35: 71–90. doi: 10.1306/74d711f4-2b21-11d7-8648000102c1865d

[12] Lighty RG. Preservation of internal reef porosity and diagenetic sealing of submerged early Holocene barrier reef, southeast Florida shelf (USA). Carbonate Cements. 1985;36: 123–151. doi: 10.2110/pec.85.36.0123

[13] Scoffin TP. Taphonomy of coral reefs: a review. Coral Reefs. 1992;11: 57–77. doi: 10.1007/BF00357423

[14] Fairbanks RG, Evans MN, Rubenstone JL, Mortlock RA, Broad K, Moore MD, et al. Evaluating climate indices and their geochemical proxies measured in corals. Coral Reefs. 1997;16: S93–S100. doi: 10.1007/s003380050245

[15] Pandolfi JM, Greenstein BJ. Preservation of community structure in death assemblages of deep-water Caribbean reef corals. Limnol Oceanogr. 1997;42: 1505–1516. doi: 10.4319/lo.1997.42.7.1505

[16] Aronson RB, Macintyre IG, Wapnick CM, O’Neill MW. Phase shifts, alternative states, and the unprecedented convergence of two reef systems. Ecology. 2004;85: 1876–1891. doi: 10.1890/03-0108

[17] Hubbard DK, Miller AI, Scaturo D. Production and cycling of calcium carbonate in a shelf-edge reef system (St. Croix, U.S. Virgin Islands): applications to the nature of reef systems in the fossil record. J Sediment Petrol. 1990;60: 335–360. doi: 10.1306/212F9197-2B24-11D7-8648000102C1865D

[18] Wood R. Taphonomy of reefs through time. In: Allison PA, and Bottjer DJ, editors. Taphonomy. Aims & Scope Topics in Geobiology Book Series. Dordrecht (Netherlands): Springer; 2011. pp. 379–409.

[19] Li C, Jones B, Blanchon P. Lagoon-shelf sediment exchange by storms-evidence from foraminiferal assemblages, east coast of Grand Cayman, British West Indies. J Sediment Res. 1997;67: 17–25. doi: 10.1306/d42684dc-2b26-11d7-8648000102c1865d

[20] Li C, Jones B, Kalbfleisch WBC. Carbonate sediment transport pathways based on foraminifera: Case study from Frank Sound, Grand Cayman, British West Indies. Sedimentology. 1998;45: 109–120. doi: 10.1046/j.1365-3091.1998.00133.x

[21] Stoddart DR. Three Caribbean atolls: Turneffe Islands, Lighthouse Reef, and Glover’s Reef, British Honduras. Atoll Res Bull. 1962;87: 1–151.

[22] Folk RL, Robles R. Carbonate Sands of Isla Perez, Alacran Reef Complex, Yucatán. J Geol. 1964;72: 255–292. doi: 10.1086/626986

[23] Perry CT, Kench PS, Smithers SG, Riegl B, Yamano H, O’Leary MJ. Implications of reef ecosystem change for the stability and maintenance of coral reef islands. Glob Change Biol. 2011;17: 3679–3696. doi: 10.1111/j.1365-2486.2011.02523.x

[24] Perry CT, Kench PS, O’Leary MJ, Morgan KM, Januchowski-Hartley F. Linking reef ecology to island building: Parrotfish identified as major producers of island-building sediment in the Maldives. Geology. 2015;43: 503–506. doi: 10.1130/G36623.1

[25] Greenstein BJ. Taphonomy: Detecting critical events in fossil reef-coral assemblages. In: Aronson RB, editor. Geological approaches to coral reef ecology. New York (USA): Springer; 2007. pp. 31–60.

[26] Greenstein BJ. Mass mortality of the West-Indian echinoid Diadema antillarum (Echinodermata: Echinoidea): a natural experiment in taphonomy. Palaios. 1989;4: 487–492. doi: 10.2307/3514593

[27] Walbran PD, Henderson RA, Timothy Jull AJ, John Head M. Evidence from sediments of long-term Acanthaster planci predation on corals of the great barrier reef. Science. 1989;245: 847–850. doi: 10.1126/science.245.4920.847 17773362

[28] Keesing JK, Bradbury RH, DeVantier LM, Riddle MJ, De’ath G. Geological evidence for recurring outbreaks of the crown-of-thorns starfish: a reassessment from an ecological perspective. Coral Reefs. 1992;11: 79–85. doi: 10.1007/BF00357425

[29] Pandolfi JM. A palaeobiological examination of the geological evidence for recurring outbreaks of the crown-of-thorns starfish, Acanthaster planci (L.). Coral Reefs. 1992;11: 87–93. doi: 10.1007/BF00357427

[30] Greenstein BJ, Pandolfi JM, Moran PJ. Taphonomy of crown-of-thorns starfish: implications for recognizing ancient population outbreaks. Coral Reefs. 1995;14: 91–97. doi: 10.1007/BF00303429

[31] Precht WF, Aronson RB. Compositional Changes in Reef Sediments Related to Changes in Coral Reef Community Structure: Abstract. Am Assoc Pet Geol Bull. 1997;81: 1561. doi: 10.1306/3b05be52-172a-11d7-8645000102c1865d

[32] Lidz BH, Hallock P. Sedimentary petrology of a declining reef ecosystem, Florida reef tract (U.S.A.). J Coast Res. 2000;16: 675–697.

[33] Goreau TF. Calcium Carbonate Deposition by Coralline Algae and Corals in Relation to Their Roles as Reef‐Builders. Ann NY Acad Sci. 1963;109: 127–167. doi: 10.1111/j.1749-6632.1963.tb13465.x 13949254

[34] Drew EA. Halimeda biomass, growth rates and sediment generation on reefs in the central great barrier reef province. Coral Reefs. 1983;2: 101–110. doi: 10.1007/BF02395280

[35] Wefer G. Carbonate production by algae Halimeda, Penicillus and Padina. Nature. 1980;285: 323–324. doi: 10.1038/285323a0

[36] Jordan E. Atlas de los arrecifes coralinos del Caribe Mexicano. Parte I. El sistema continental. Centro de Investigaciones de Quintana Roo (CIQRO)/Instituto de Ciencias del Mar y Limnología, UNAM, México. 1993.

[37] Perry CT, Lange ID. ReefBudget Caribbean v2: online resource and methodology. In: http://geography.exeter.ac.uk/reefbudget/ [Internet]. 2019.

[38] González-Barrios FJ, Álvarez-Filip L. A framework for measuring coral species-specific contribution to reef functioning in the Caribbean. Ecol Indic. 2018;95: 877–886. doi: 10.1016/j.ecolind.2018.08.038

[39] Griffin SP, García RP, Weil E. Bioerosion in coral reef communities in southwest Puerto Rico by the sea urchin Echinometra viridis. Mar Biol. 2003;143: 79–84. doi: 10.1007/s00227-003-1056-1

[40] Scoffin TP, Stearn CW, Boucher D, Frydl P, Hawkins CM, Hunter IG, et al. Calcium carbonate budget of a fringing reef on the west coast of Barbados: Part II, Erosion, sediments and internal structure. Bull Mar Sci. 1980;30: 475–508.

[41] Brown-Saracino J, Peckol P, Allen Curran H, Robbart ML. Spatial variation in sea urchins, fish predators, and bioerosion rates on coral reefs of Belize. Coral Reefs. 2007;26: 71–78. doi: 10.1007/s00338-006-0159-9

[42] de Bakker DM, Webb AE, van den Bogaart LA, Van Heuven SMAC, Meesters EH, van Duyl FC. Quantification of chemical and mechanical bioerosion rates of six Caribbean excavating sponge species found on the coral reefs of Curaçao. PLoS One. 2018;13: e0197824. doi: 10.1371/journal.pone.0197824 29847572 PMC5976196

[43] Bruggemann JH, Van Kessel AM, Van Rooij JM, Breeman AM. Bioerosion and sediment ingestion by the caribbean parrotfish Scarus vetula and Sparisoma viride: Implications of fish size, feeding mode and habitat use. Mar Ecol Prog Ser. 1996;134: 59–71. doi: 10.3354/meps134059

[44] Mumby PJ. The impact of exploiting grazers (Scaridae) on the dynamics of Caribbean coral reefs. Ecol Appl. 2006;16: 747–769. doi: 10.1890/1051-0761(2006)016[0747:tioegs]2.0.co;2 16711060

[45] Bellwood DR. Direct estimate of bioerosion by two parrotfish species, Chlorurus gibbus and C. sordidus, on the Great Barrier Reef, Australia. Mar Biol. 1995;121: 419–429. doi: 10.1007/BF00349451

[46] Perry CT, Alvarez-Filip L, Graham NAJ, Mumby PJ, Wilson SK, Kench PS, et al. Loss of coral reef growth capacity to track future increases in sea level. Nature. 2018;558: 396–400. doi: 10.1038/s41586-018-0194-z 29904103

[47] van Woesik R, Cacciapaglia CW. Carbonate production of Micronesian reefs suppressed by thermal anomalies and Acanthaster as sea-level rises. PLoS One. 2019;14: e0224887. doi: 10.1371/journal.pone.0224887 31730649 PMC6857905

[48] van Woesik R, Cacciapaglia CW. Keeping up with sea-level rise: Carbonate production rates in Palau and Yap, western Pacific Ocean. PLoS One. 2018;13: e0197077. doi: 10.1371/journal.pone.0197077 29738545 PMC5940225

[49] Hughes TP, Baird AH, Dinsdale EA, Moltschaniwskyj NA, Pratchett MS, Tanner JE, et al. Patterns of recruitment and abundance of corals along the Great Barrier Reef. Nature. 1999;397: 59–63. doi: 10.1038/16237

[50] Murdoch TJT, Aronson RB. Scale-dependent spatial variability of coral assemblages along the Florida Reef Tract. Coral Reefs. 1999;18: 341–351. doi: 10.1007/s003380050210

[51] Goreau TF, Goreau NI. Coral Reef Project—Papers in Memory of Dr. Thomas F. Goreau. 17. The Ecology of Jamaican Coral Reefs. II. Geomorphology, Zonation, and Sedimentary Phases. Bull Mar Sci. 1973;23.

[52] Yamano H, Kayanne H, Chikamori M. An Overview of The Nature and Dynamics of Reef Islands. Global Environ Res. 2005;9: 9–20.

[53] Oksanen J, Simpson Gavin, Blanchet F, Kindt Roeland, Legendre Pierre, Minchin Peter, et al. vegan: Community Ecology Package. R package version 2.6–4. Community ecology package. 2022;2.6–4. https://CRAN.Rproject.org/package=vegan.

[54] Kindt R, Coe R. Tree diversity analysis; A manual and software for common statistical methods for ecological and biodiversity studies. World Agroforestry Centre (ICRAF), Nairobi (Kenya). 2005. ISBN 92-9059-179-X. http://www.worldagroforestry.org/output/treediversity-analysis.

[55] Pinheiro J, Bates D, DebRoy S, Sarkar D. R Core Team (2014). nlme: linear and nonlinear mixed effects models. R package version 3.1–117. 2021. https://cran.r-project.org/package=nlme.R-project.

[56] R Development Core Team. R Core Team. R: A language and environment for statistical computing. R Foundation for Statistical Computing, Vienna, Austria. 2022. https://www.R-project.org/. Vol. 2, R Foundation for Statistical Computing.

[57] Molina-Hernández A, González-Barrios FJ, Perry CT, Álvarez-Filip L. Two decades of carbonate budget change on shifted coral reef assemblages: Are these reefs being locked into low net budget states?: Caribbean reefs carbonate budget trends. P R Soc B. 2020;287: 20202305. doi: 10.1098/rspb.2020.2305 33290684 PMC7739932

[58] Perry CT, Morgan KM, Lange ID, Yarlett RT. Bleaching-driven reef community shifts drive pulses of increased reef sediment generation. R Soc Open Sci. 2020;7: 192153. doi: 10.1098/rsos.192153 32431891 PMC7211869

[59] Perry CT, Murphy GN, Kench PS, Smithers SG, Edinger EN, Steneck RS, et al. Caribbean-wide decline in carbonate production threatens coral reef growth. Nat Commun. 2013;4: 1402. doi: 10.1038/ncomms2409 23360993 PMC3660652

[60] Perry CT, Steneck RS, Murphy GN, Kench PS, Edinger EN, Smithers SG, et al. Regional-scale dominance of non-framework building corals on Caribbean reefs affects carbonate production and future reef growth. Glob Chang Biol. 2015;21: 1153–1164. doi: 10.1111/gcb.12792 25537577

[61] Adam TC, Duran A, Fuchs CE, Roycroft M V., Rojas MC, Ruttenberg BI, et al. Comparative analysis of foraging behavior and bite mechanics reveals complex functional diversity among Caribbean parrotfishes. Mar Ecol Prog Ser. 2018;597: 207–220. doi: 10.3354/meps12600

[62] Bellwood DR, Choat JH. A functional analysis of grazing in parrotfishes (family Scaridae): the ecological implications. Environ Biol Fishes. 1990;28: 189–214. doi: 10.1007/BF00751035

[63] Yarlett RT, Perry CT, Wilson RW, Harborne AR. Inter-habitat variability in parrotfish bioerosion rates and grazing pressure on an Indian Ocean reef platform. Diversity. 2020;12: 381. doi: 10.3390/d12100381

[64] Medina-Valmaseda AE, Rodríguez-Martínez RE, Alvarez-Filip L, Jordan-Dahlgren E, Blanchon P. The role of geomorphic zonation in long-term changes in coral-community structure on a Caribbean fringing reef. PeerJ. 2020;8: e10103. doi: 10.7717/peerj.10103 33150066 PMC7585725

[65] Lighty RG, Macintyre IG, Stuckenrath R. Acropora palmata reef framework: A reliable indicator of sea level in the western atlantic for the past 10,000 years. Coral Reefs. 1982;1: 125–130. doi: 10.1007/BF00301694

[66] Blanchon P, Richards S, Bernal JP, Cerdeira-Estrada S, Ibarra MS, Corona-Martínez L, et al. Retrograde accretion of a caribbean fringing reef controlled by hurricanes and sea-level rise. Front Earth Sci. 2017;5: 78. doi: 10.3389/feart.2017.00078

[67] Toth LT, Stathakopoulos A, Kuffner IB, Ruzicka RR, Colella MA, Shinn EA. The unprecedented loss of Florida’s reef-building corals and the emergence of a novel coral-reef assemblage. Ecology. 2019;100: e02781. doi: 10.1002/ecy.2781 31170313 PMC6851685

[68] Vecsei A. Fore-reef carbonate production: Development of a regional census-based method and first estimates. Palaeogeogr Palaeoecol. 2001;175: 185–200. doi: 10.1016/S0031-0182(01)00371-6

[69] Aronson RB, Precht WF. White-band disease and the changing face of Caribbean coral reefs. In: Porter JW, editor. The Ecology and Etiology of Newly Emerging Marine Diseases. Dordrecht (Netherlands): Springer; 2001. pp. 25–38.

[70] Bruckner AW, Bruckner RJ. The recent decline of Montastraea annularis (complex) coral populations in western Curaçao: A cause for concern? Rev Biol Trop. 2006;54: 45–58.

[71] Edmunds PJ, Elahi R. The demographics of a 15-year decline in cover of the Caribbean reef coral Montastraea annularis. Ecol Monogr. 2007;77: 3–18. doi: 10.1890/05-1081

[72] Lessios HA, Robertson DR, Cubit JD. Spread of Diadema mass mortality through the Caribbean. Science. 1984;226: 335–337. doi: 10.1126/science.226.4672.335 17749884

[73] Glynn PW. El Niño warming, coral mortality and reef framework destruction by echinoid bioerosion in the eastern Pacific. Galaxea. 1988;7: 129–160.

[74] Eakin CM. A tale of two enso events: Carbonate budgets and the influence of two warming disturbances and intervening variability, Uva Island, Panama. Bull Mar Sci. 2001;69: 171–186.

[75] Eakin CM. Where have all the carbonates gone? A model comparison of calcium carbonate budgets before and after the 1982–1983 El Niño at Uva Island in the eastern Pacific. Coral Reefs. 1996;15: 109–119. doi: 10.1007/s003380050031

[76] Glynn PW. Widespread Coral Mortality and the 1982–83 El Niño Warming Event. Environ Conserv. 1984;11: 133–146. doi: 10.1017/S0376892900013825

[77] Hernández-Ballesteros LM, Elizalde-Rendón EM, Carballo JL, Carricart-Ganivet JP. Sponge bioerosion on reef-building corals: Dependent on the environment or on skeletal density? J Exp Mar Biol Ecol. 2013;441: 23–27. doi: 10.1016/j.jembe.2013.01.016

[78] Molina-Hernández A, Medellín-Maldonado F, Lange ID, Perry CT, Álvarez-Filip L. Coral reef erosion: In situ measurement on different dead coral substrates on a Caribbean reef. Limnol Oceanogr. 2022;67: 2734–2749. doi: 10.1002/lno.12234

[79] Kuffner IB, Toth LT, Hudson JH, Goodwin WB, Stathakopoulos A, Bartlett LA, et al. Improving estimates of coral reef construction and erosion with in situ measurements. Limnol Oceanogr. 2019;64: 2283–2294. doi: 10.1002/lno.11184

[80] Russ GR, Questel SLA, Rizzari JR, Alcala AC. The parrotfish—coral relationship: refuting the ubiquity of a prevailing paradigm. Mar Biol. 2015;162: 2029–2045. doi: 10.1007/s00227-015-2728-3

[81] Burman SG, Aronson RB, van Woesik R. Biotic homogenization of coral assemblages along the Florida reef tract. Mar Ecol Prog Ser. 2012;467: 89–96. doi: 10.3354/meps09950

[82] Gilmour JP, Smith LD, Heyward AJ, Baird AH, Pratchett MS. Recovery of an isolated coral reef system following severe disturbance. Science. 2013;340: 69–71. doi: 10.1126/science.1232310 23559247

[83] Graham NAJ, Jennings S, MacNeil MA, Mouillot D, Wilson SK. Predicting climate-driven regime shifts versus rebound potential in coral reefs. Nature. 2015;518: 94–97. doi: 10.1038/nature14140 25607371

[84] Edmunds PJ, Adam TC, Baker AC, Doo SS, Glynn PW, Manzello DP, et al. Why more comparative approaches are required in time-series analyses of coral reef ecosystems. Mar Ecol Prog Ser. 2019;608: 297–306. doi: 10.3354/meps12805

[85] Fowler AJ, Doherty PJ, Williams DMB. Multi-scale analysis of recruitment of a coral reef fish on the Great Barrier Reef. Mar Ecol Prog Ser. 1992;82: 131–141. doi: 10.3354/meps082131

[86] Williams SM, Chollett I, Roff G, Cortés J, Dryden CS, Mumby PJ. Hierarchical spatial patterns in Caribbean reef benthic assemblages. J Biogeogr. 2015;42: 1327–1335. doi: 10.1111/jbi.12509

[87] Rodriguez-Ruano V, Toth LT, Enochs IC, Randall CJ, Aronson RB. Upwelling, climate change, and the shifting geography of coral reef development. Sci Rep. 2023;13: 1770. doi: 10.1038/s41598-023-28489-0 36750639 PMC9905564

[88] Gardner TA, Côté IM, Gill JA, Grant A, Watkinson AR. Long-term region-wide declines in Caribbean corals. Science. 2003;301: 958–960. doi: 10.1126/science.1086050 12869698

[89] Contreras-Silva AI, Tilstra A, Migani V, Thiel A, Pérez-Cervantes E, Estrada-Saldívar N, et al. A meta-analysis to assess long-term spatiotemporal changes of benthic coral and macroalgae cover in the Mexican Caribbean. Sci Rep. 2020;10: 8897. doi: 10.1038/s41598-020-65801-8 32483234 PMC7264131

[90] Perry CT. Factors controlling sediment preservation on a north Jamaican fringing reef: A process-based approach to microfacies analysis. J Sediment Res. 2000;70: 633–648. doi: 10.1306/2DC4092D-0E47-11D7-8643000102C1865D

