# Supplementary figures and images for: Spatial variability of sedimentary assemblages reflects variations in bioerosion pressure of adjacent coral reefs
Source: PLoS One. 2024 Oct 11;19(10):e0311344. doi: 10.1371/journal.pone.0311344 (PMC11469488; doi:10.1371/journal.pone.0311344)

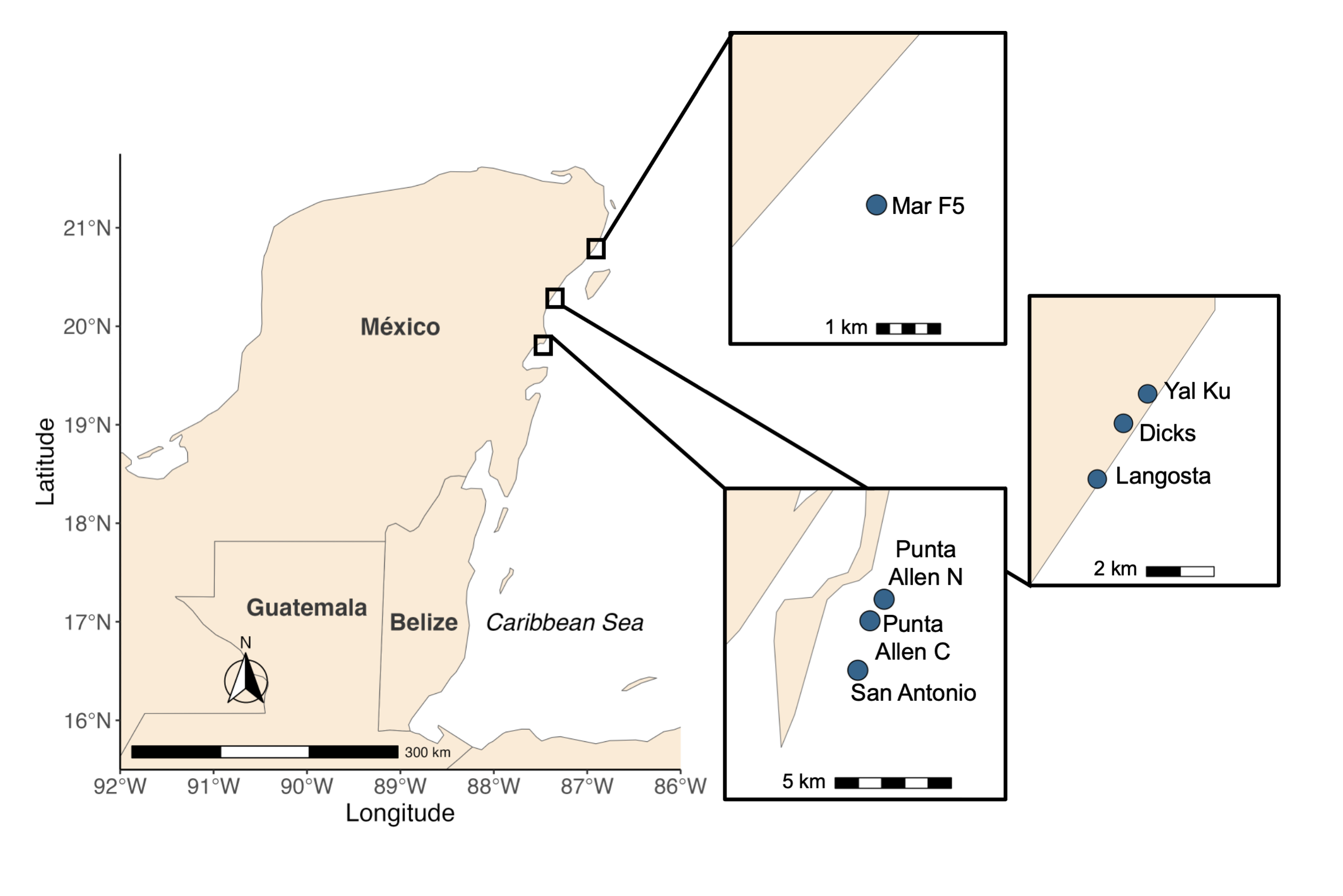

Supplement: S1 Fig — The study sites within localities are shown as insets. These maps were generated using Natural Earth Data (http://www.naturalearthdata.com/), which were retrieved using the “rnaturalearth” package in R version 4.2.2. (TIF) [file pone.0311344.s001.tif]

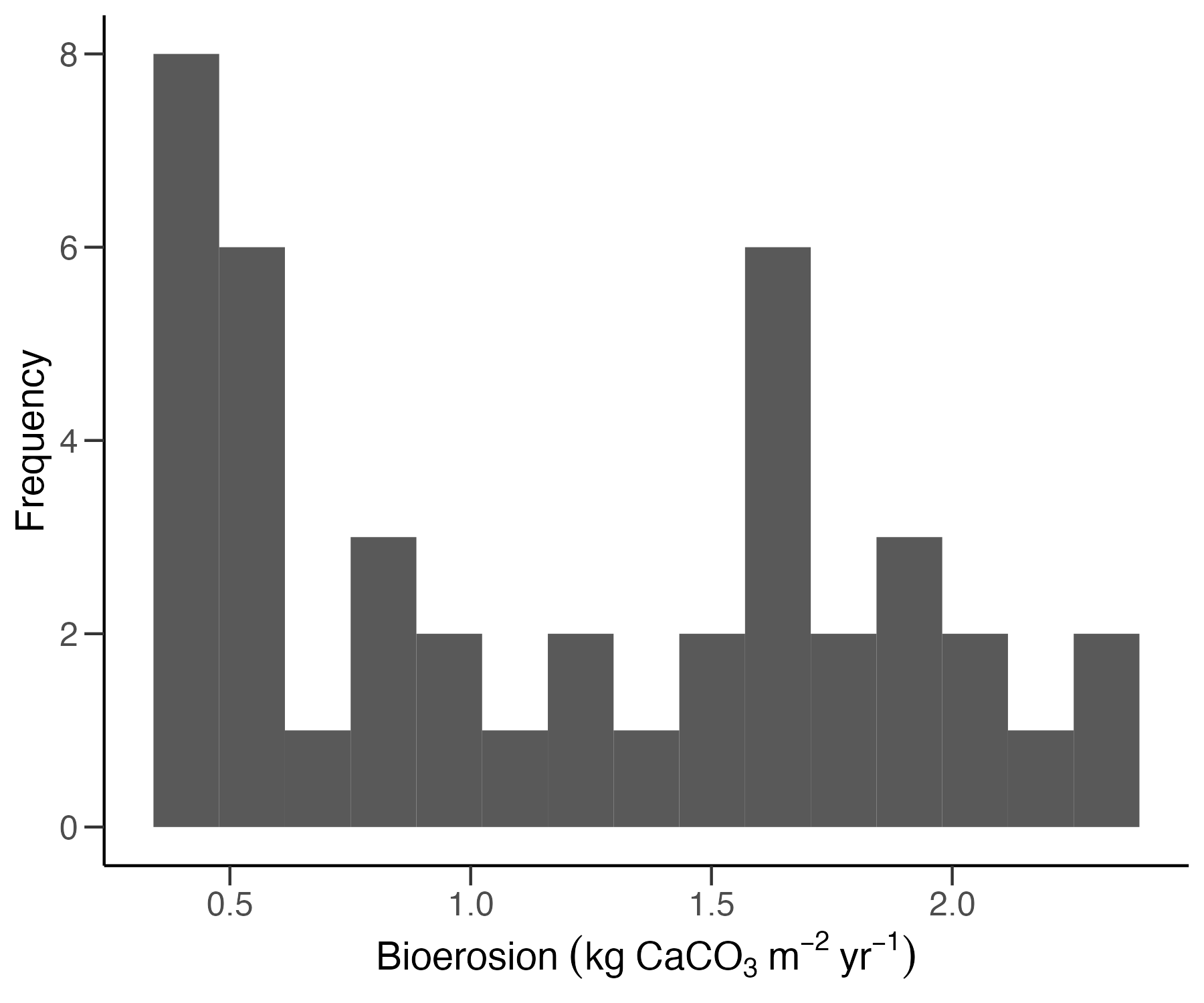

Supplement: S2 Fig — (TIFF) [file pone.0311344.s002.tiff]

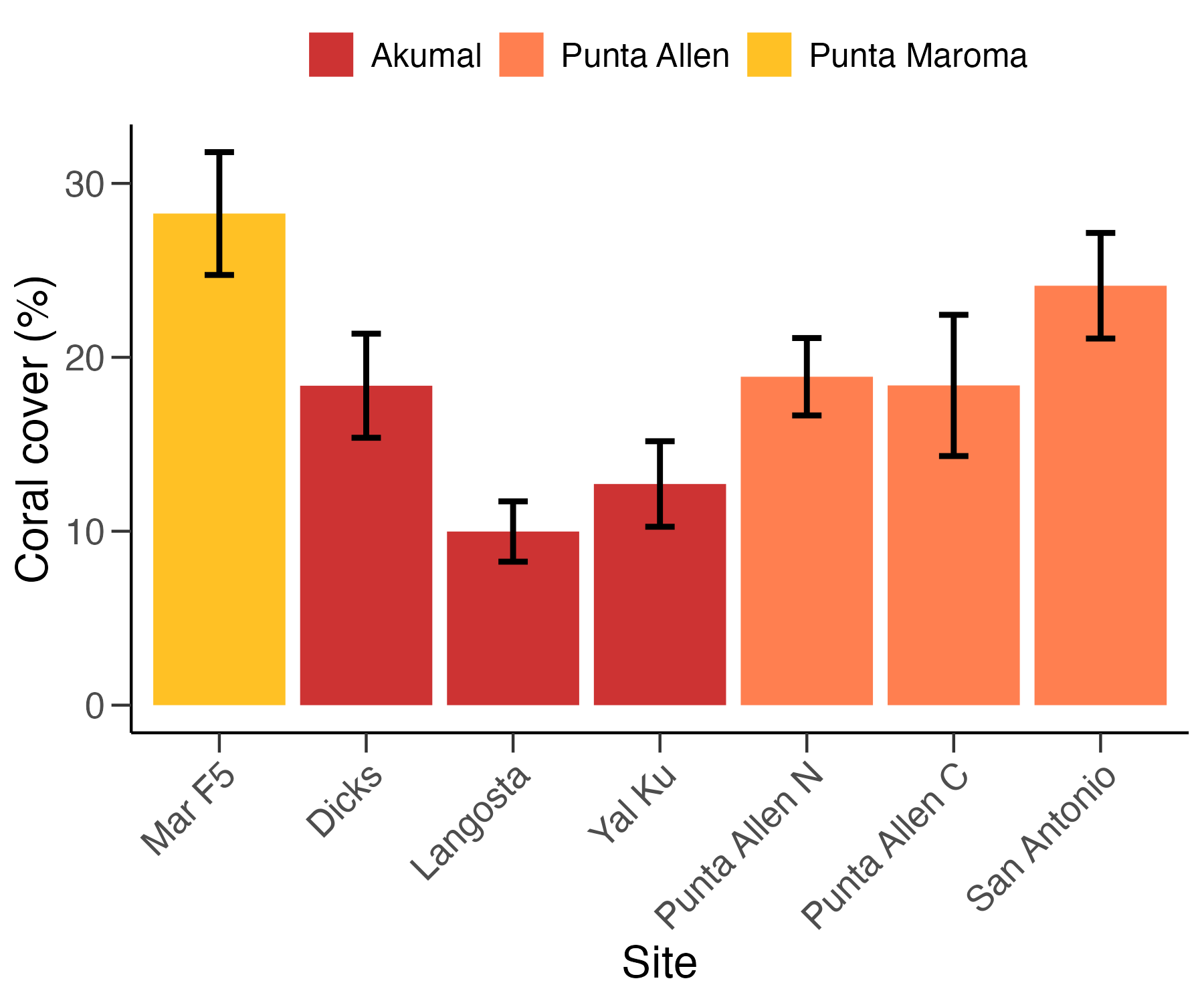

Supplement: S3 Fig — Bars represent the mean (± standard error) percent coral cover at each site we surveyed. Colors denote the locality for each site. (TIFF) [file pone.0311344.s003.tiff]

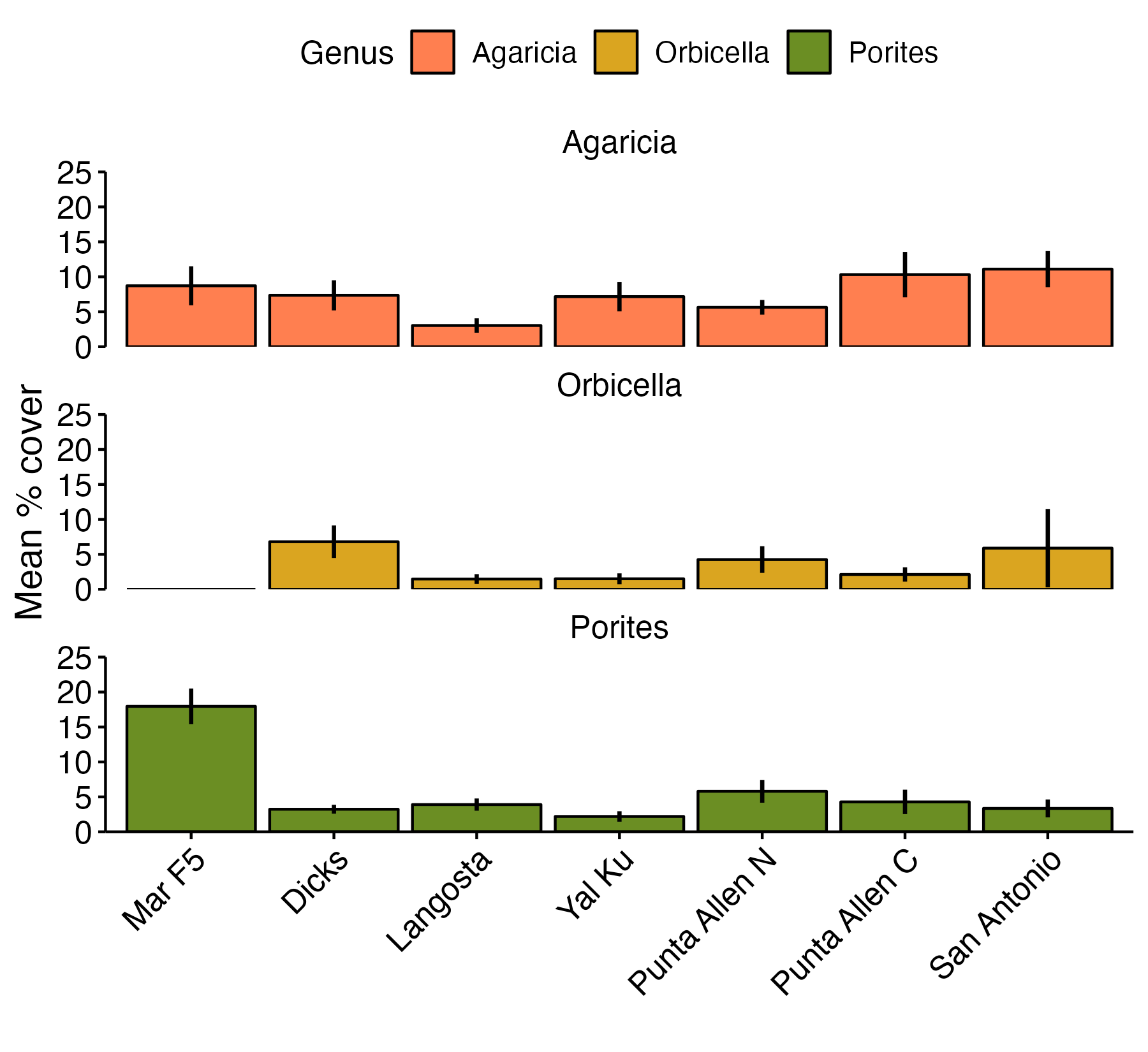

Supplement: S4 Fig — Bars represent the mean (± standard error) percent cover estimates for the dominant weedy (Agaricia and Porites) and framework-building (Orbicella) coral taxa at each site. Punta Allen N = Punta Allen Norte, Punta Allen C = Punta Allen Centro. (TIFF) [file pone.0311344.s004.tiff]

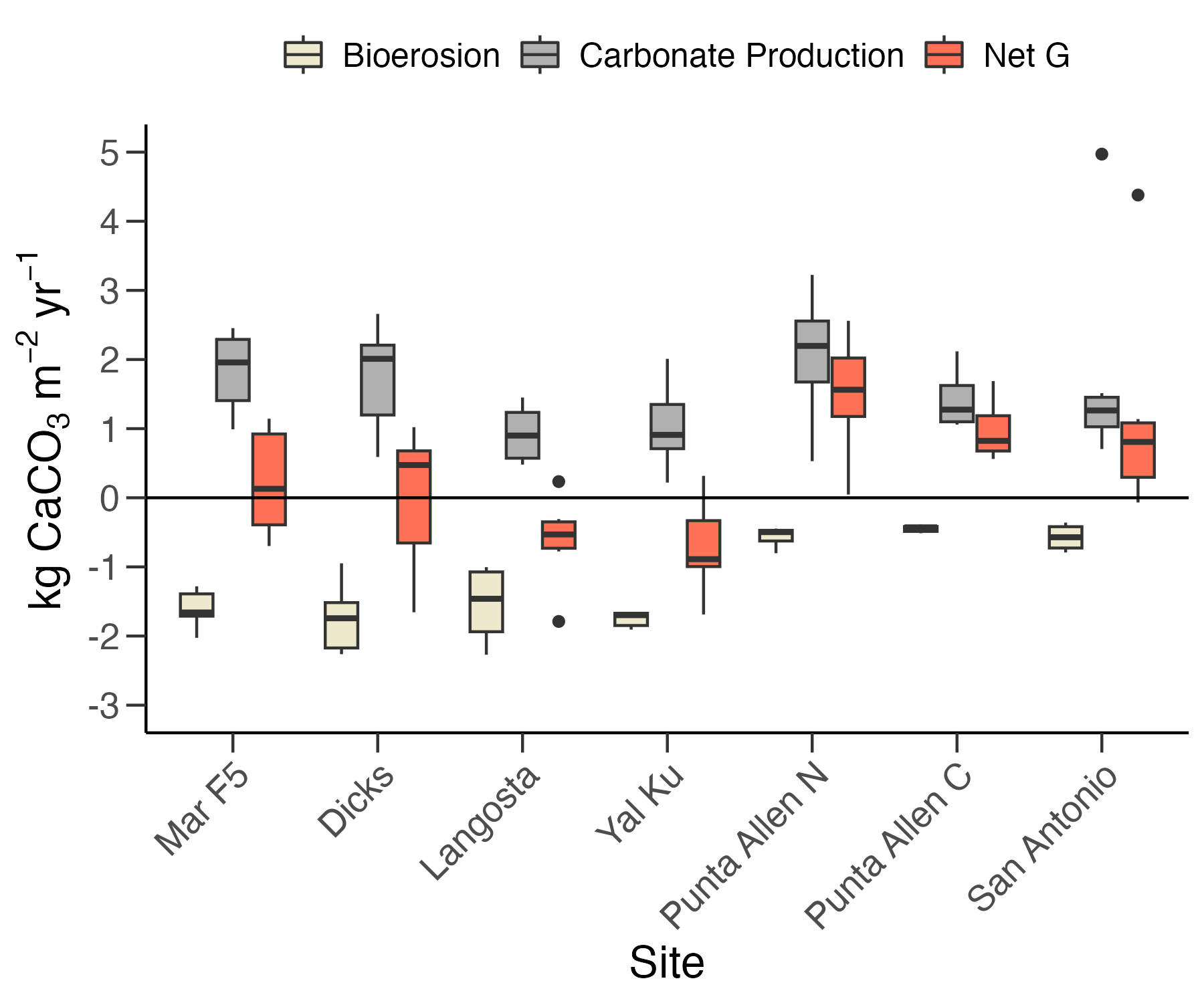

Supplement: S5 Fig — Boxplots represent the median (± interquartile range) total bioerosion (tan), gross carbonate production (gray), and net carbonate production (red) for each site. All of the rates are reported in kg CaCO3 m-2 yr-1. The black horizontal line delimits net production and net erosion. Black points represent statistical outliers. (TIFF) [file pone.0311344.s005.tiff]

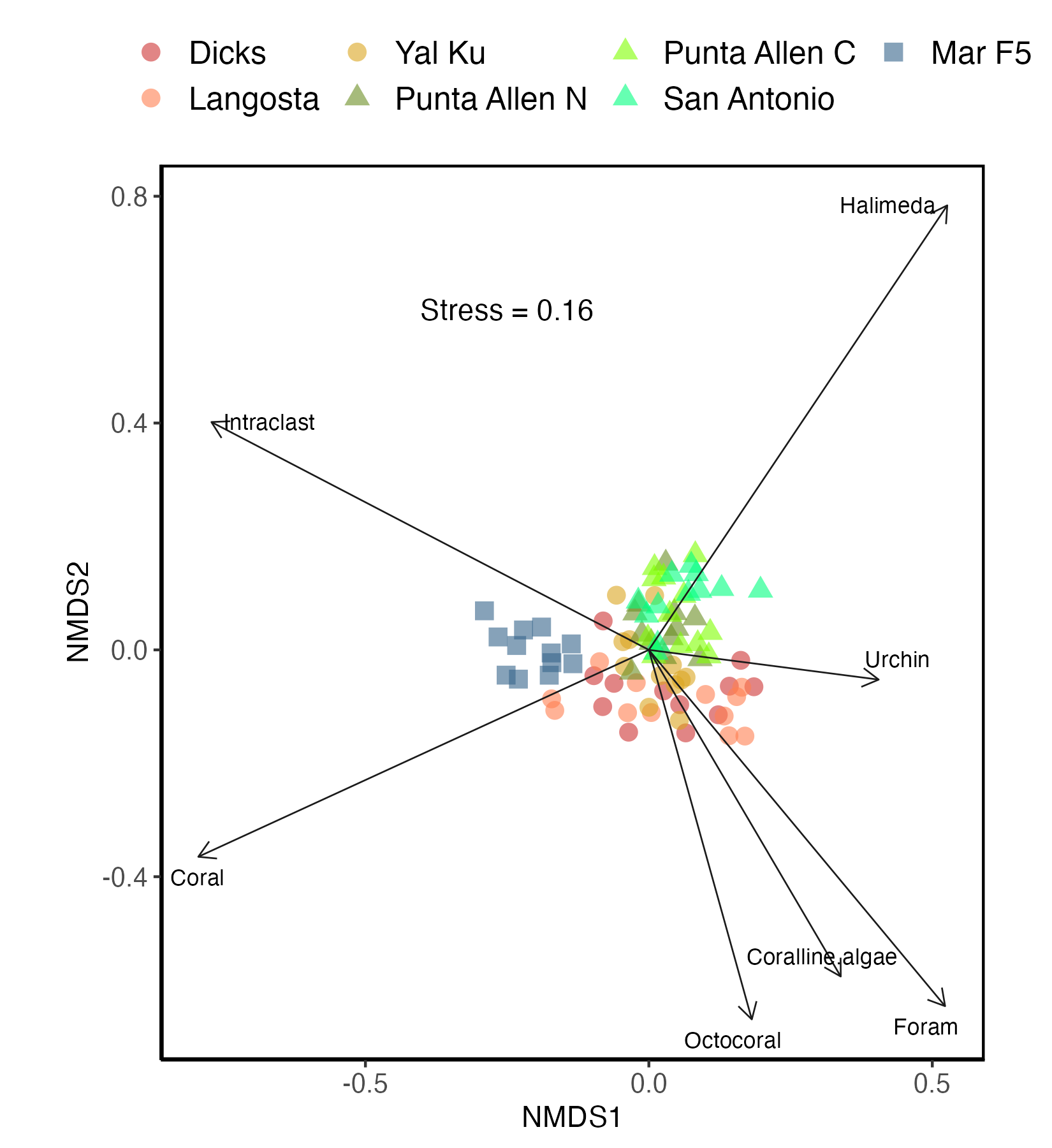

Supplement: S6 Fig — The non-metric multidimensional scaling (nMDS) plot shows the relationship of sediment samples to the separation among sites. The species vectors shown are the sedimentary categories that contribute the most to the separation among sites. Squares represent the sites from Punta Maroma, circles represent the sites from Akumal, and triangles represent the sites form Punta Allen. (TIFF) [file pone.0311344.s006.tiff]
