# Supplementary material for: Spatial variability of sedimentary assemblages reflects variations in bioerosion pressure of adjacent coral reefs
Source: PLoS One. 2024 Oct 11;19(10):e0311344. doi: 10.1371/journal.pone.0311344 (PMC11469488; doi:10.1371/journal.pone.0311344)
Supplement: S1 Table — Nested ANOVA results testing for differences in mean coral cover (%) among localities and the sites nested within them. (DOCX) [file pone.0311344.s007.docx]

**S1 Table. Variation in coral cover across spatial scales.** Nested ANOVA results testing for differences in mean coral cover (%) among localities and the sites nested within them.

| **Source** | **DF** | **Sum of Squares** | **Mean Square** | **F** | **p** |
| --- | --- | --- | --- | --- | --- |
| Locality | 2 | 1056.9 | 528.5 | 6.211 | 0.06 |
| Site{Locality} | 4 | 340.4 | 85.1 | 1.624 | 0.19 |
| Error | 35 | 1833.6 | 52.4 |  |  |
| Total | 41 | 3230.9 |  |  |  |
