# Supplementary material for: Spatial variability of sedimentary assemblages reflects variations in bioerosion pressure of adjacent coral reefs
Source: PLoS One. 2024 Oct 11;19(10):e0311344. doi: 10.1371/journal.pone.0311344 (PMC11469488; doi:10.1371/journal.pone.0311344)
Supplement: S2 Table — Nested ANOVA results testing for differences in mean gross carbonate production (kg CaCO3 m-2 yr-1) among localities and the sites nested within them. (DOCX) [file pone.0311344.s008.docx]

**S2 Table. Variation in carbonate production across spatial scales.** Nested ANOVA results testing for differences in mean gross carbonate production (kg CaCO_3_ m^-2^ yr^-1^) among localities and the sites nested within them.

| **Source** | **DF** | **Sum of Squares** | **Mean Square** | **F** | **p** |
| --- | --- | --- | --- | --- | --- |
| Locality | 2 | 1.91 | 0.95 | 2.20 | 0.23 |
| Site{Locality} | 4 | 1.73 | 0.43 | 1.34 | 0.27 |
| Error | 35 | 11.33 | 0.3238 |  |  |
| Total | 41 | 14.97 |  |  |  |
