# Supplementary material for: Spatial variability of sedimentary assemblages reflects variations in bioerosion pressure of adjacent coral reefs
Source: PLoS One. 2024 Oct 11;19(10):e0311344. doi: 10.1371/journal.pone.0311344 (PMC11469488; doi:10.1371/journal.pone.0311344)
Supplement: S3 Table — Tukey HSD post-hoc, pairwise comparisons of mean bioerosion rates of all localities. Significant comparisons are highlighted in gray. (DOCX) [file pone.0311344.s009.docx]

**S3 Table. Post-hoc, pairwise comparisons of bioerosion rates across spatial scales.** Tukey HSD post-hoc, pairwise comparisons of mean bioerosion rates of all localities. Significant comparisons are highlighted in gray.

| **Pairs** | **Difference** | **Lower Interval** | **Upper Interval** | **p** |
| --- | --- | --- | --- | --- |
| Punta Allen-Akumal | -1.15 | -1.40 | -0.90 | 0.000*** |
| Punta Maroma-Akumal | -0.07 | -0.42 | 0.29 | 0.88 |
| Punta Maroma-Punta Allen | 1.08 | 0.73 | 1.44 | 0.000*** |
