# Supplementary material for: Spatial variability of sedimentary assemblages reflects variations in bioerosion pressure of adjacent coral reefs
Source: PLoS One. 2024 Oct 11;19(10):e0311344. doi: 10.1371/journal.pone.0311344 (PMC11469488; doi:10.1371/journal.pone.0311344)
Supplement: S4 Table — Nested ANOVA results testing for differences in mean net carbonate production (kg CaCO3 m-2 yr-1) among localities and the sites nested within them. Significant results are highlighted in gray. (DOCX) [file pone.0311344.s010.docx]

**S4 Table. Variation in net carbonate production across spatial scales.** Nested ANOVA results testing for differences in mean net carbonate production (kg CaCO_3_ m^-2^ yr^-1^) among localities and the sites nested within them. Significant results are highlighted in gray.

| **Source** | **DF** | **Sum of Squares** | **Mean Square** | **F** | **p** |
| --- | --- | --- | --- | --- | --- |
| Locality | 2 | 25.16 | 12.58 | 18.91 | 0.009** |
| Site{Locality} | 4 | 2.66 | 0.67 | 0.74 | 0.57 |
| Error | 35 | 31.52 | 0.90 |  |  |
| Total | 41 | 59.34 |  |  |  |
