# Supplementary material for: Spatial variability of sedimentary assemblages reflects variations in bioerosion pressure of adjacent coral reefs
Source: PLoS One. 2024 Oct 11;19(10):e0311344. doi: 10.1371/journal.pone.0311344 (PMC11469488; doi:10.1371/journal.pone.0311344)
Supplement: S5 Table — Tukey HSD post-hoc, pairwise comparisons of mean net carbonate production rates of all localities. Significant comparisons are highlighted in gray. (DOCX) [file pone.0311344.s011.docx]

**S5 Table. Post-hoc, pairwise comparisons of net carbonate production rates across spatial scales.** Tukey HSD post-hoc, pairwise comparisons of mean net carbonate production rates of all localities. Significant comparisons are highlighted in gray.

| **Pairs** | **Difference** | **Lower Interval** | **Upper Interval** | **p** |
| --- | --- | --- | --- | --- |
| Punta Allen-Akumal | 1.67 | 0.91 | 2.43 | 0.000*** |
| Punta Maroma-Akumal | 0.67 | -0.41 | 1.74 | 0.30 |
| Punta Maroma-Punta Allen | -1.00 | -2.08 | 0.07 | 0.07 |
