# Supplementary material for: Spatial variability of sedimentary assemblages reflects variations in bioerosion pressure of adjacent coral reefs
Source: PLoS One. 2024 Oct 11;19(10):e0311344. doi: 10.1371/journal.pone.0311344 (PMC11469488; doi:10.1371/journal.pone.0311344)
Supplement: S6 Table — Nested PERMANOVA results testing the degree of separation of sedimentary assemblages among localities and the sites nested within them. Significant results are highlighted in gray. (DOCX) [file pone.0311344.s012.docx]

**S6 Table. Variation in sedimentary assemblages across spatial scales.** Nested PERMANOVA results testing the degree of separation of sedimentary assemblages among localities and the sites nested within them. Significant results are highlighted in gray.

| **Source** | **DF** | **Sum of Squares** | **R^2^** | **F** | **p** |
| --- | --- | --- | --- | --- | --- |
| Locality | 2 | 0.32849 | 0.40 | 28.9090 | 0.0001*** |
| Site{Locality} | 4 | 0.05444 | 0.07 | 2.3953 | 0.0044** |
| Error | 77 | 0.43748 | 0.53 |  |  |
| Total | 83 | 0.82041 | 1.00 |  |  |
