# Supplementary material for: Spatial variability of sedimentary assemblages reflects variations in bioerosion pressure of adjacent coral reefs
Source: PLoS One. 2024 Oct 11;19(10):e0311344. doi: 10.1371/journal.pone.0311344 (PMC11469488; doi:10.1371/journal.pone.0311344)
Supplement: S7 Table — Contrast tables of pairwise comparisons from the similarity of percentages (SIMPER) test on the abundance of sediment categories found for each site. The rows highlighted in gray indicate variables that were significantly different for the respective site pairs. (DOCX) [file pone.0311344.s013.docx]

**S7 Table. Post-hoc, pairwise comparisons of sedimentary assemblages across spatial scales.** Contrast tables of pairwise comparisons from the similarity of percentages (SIMPER) test on the abundance of sediment categories found for each site. The rows highlighted in gray indicate variables that were significantly different for the respective site pairs.

| **Dicks–Langosta** | | | | | | | | | | |
| --- | --- | --- | --- | --- | --- | --- | --- | --- | --- | --- |
|  | **Average** | **SD** | **Ratio** | **Ava** | **Avb** | **Cum. sum** | | **p** | |  |
| Coral | 0.022397 | 0.016225 | 1.3804 | 0.6019 | 0.5794 | 0.175 | | 0.0098** | |  |
| Foram | 0.017965 | 0.012731 | 1.4112 | 0.3415 | 0.35 | 0.315 | | 0.2368 | |  |
| Coralline algae | 0.017379 | 0.012825 | 1.3551 | 0.4534 | 0.4573 | 0.451 | | 0.0072** | |  |
| Urchin | 0.013069 | 0.009369 | 1.395 | 0.1507 | 0.1407 | 0.553 | | 0.1256 | |  |
| *Homotrema* | 0.012972 | 0.009074 | 1.4296 | 0.1252 | 0.0859 | 0.655 | | 0.1976 | |  |
| Mollusk | 0.012877 | 0.009746 | 1.3213 | 0.3619 | 0.3551 | 0.755 | | 0.8962 | |  |
| Intraclast | 0.012811 | 0.007796 | 1.6432 | 0.2318 | 0.2864 | 0.856 | | 0.0045** | |  |
| *Halimeda* | 0.011489 | 0.008786 | 1.3077 | 0.3478 | 0.3307 | 0.945 | | 1 | |  |
| Octocoral | 0.007008 | 0.005028 | 1.3939 | 0.1338 | 0.1518 | 1 | | 0.9415 | |  |
| **Dicks–Yal Ku** | | | | | | | | | | |
|  | **Average** | **SD** | **Ratio** | **Ava** | **Avb** | | **Cum. sum** | | **p** |  |
| Foram | 0.017507 | 0.008614 | 2.0325 | 0.6019 | 0.5802 | | 0.147 | | 0.5623 |  |
| *Homotrema* | 0.016388 | 0.011077 | 1.4795 | 0.3415 | 0.291 | | 0.284 | | 0.4937 |  |
| Coral | 0.014706 | 0.010529 | 1.3967 | 0.1252 | 0.0961 | | 0.407 | | 0.0204* |  |
| Mollusk | 0.013966 | 0.009937 | 1.4054 | 0.4534 | 0.4716 | | 0.524 | | 0.2338 |  |
| Urchin | 0.013462 | 0.008538 | 1.5768 | 0.2318 | 0.2898 | | 0.637 | | 0.0012** |  |
| Coralline algae | 0.013095 | 0.010994 | 1.1911 | 0.1507 | 0.0894 | | 0.747 | | 0.121 |  |
| *Halimeda* | 0.01171 | 0.007887 | 1.4847 | 0.3478 | 0.3896 | | 0.845 | | 1 |  |
| Intraclast | 0.009818 | 0.007107 | 1.3814 | 0.3619 | 0.385 | | 0.927 | | 0.9927 |  |
| Octocoral | 0.00872 | 0.007502 | 1.1624 | 0.1338 | 0.1016 | | 1 | | 0.6387 |  |
| **Dicks–San Antonio** | | | | | | | | | | |
|  | **Average** | **SD** | **Ratio** | **Ava** | **Avb** | | **Cum. sum** | | **p** |  |
| *Halimeda* | 0.03763 | 0.016637 | 2.2619 | 0.3478 | 0.5522 | | 0.253 | | 0.0023** |  |
| Foram | 0.02475 | 0.015126 | 1.6359 | 0.3415 | 0.2161 | | 0.42 | | 0.0003*** |  |
| Coral | 0.02292 | 0.015196 | 1.5083 | 0.6019 | 0.5132 | | 0.574 | | 0.0053** |  |
| Coralline algae | 0.01466 | 0.010244 | 1.4314 | 0.4534 | 0.4252 | | 0.673 | | 0.1432 |  |
| *Homotrema* | 0.01041 | 0.008227 | 1.265 | 0.1252 | 0.1034 | | 0.743 | | 0.7884 |  |
| Mollusk | 0.01033 | 0.007792 | 1.3258 | 0.3619 | 0.4026 | | 0.813 | | 0.9885 |  |
| Intraclast | 0.01025 | 0.00726 | 1.4117 | 0.2318 | 0.2436 | | 0.882 | | 0.3575 |  |
| Urchin | 0.009 | 0.007517 | 1.1976 | 0.1507 | 0.1442 | | 0.942 | | 0.9074 |  |
| Octocoral | 0.00857 | 0.007581 | 1.1308 | 0.1338 | 0.0953 | | 1 | | 0.6816 |  |
| **Dicks–Mar F5** | | | | | | | | | | |
|  | **Average** | **SD** | **Ratio** | **Ava** | **Avb** | | **Cum. sum** | | **p** |  |
| Intraclast | 0.03694 | 0.010488 | 3.522 | 0.3619 | 0.5595 | | 0.211 | | 0.0001*** |  |
| *Halimeda* | 0.03274 | 0.01141 | 2.869 | 0.3478 | 0.1728 | | 0.398 | | 0.0804 |  |
| Foram | 0.02574 | 0.015804 | 1.629 | 0.3415 | 0.2093 | | 0.545 | | 0.0001*** |  |
| Coral | 0.01849 | 0.012491 | 1.481 | 0.6019 | 0.6573 | | 0.651 | | 0.3595 |  |
| Coralline algae | 0.01541 | 0.010937 | 1.409 | 0.4534 | 0.396 | | 0.739 | | 0.0743 |  |
| Urchin | 0.01234 | 0.010348 | 1.192 | 0.1507 | 0.118 | | 0.809 | | 0.2424 |  |
| Mollusk | 0.01211 | 0.007923 | 1.528 | 0.2318 | 0.2785 | | 0.879 | | 0.0207* |  |
| *Homotrema* | 0.01139 | 0.008823 | 1.291 | 0.1252 | 0.111 | | 0.944 | | 0.5742 |  |
| Octocoral | 0.00986 | 0.007536 | 1.309 | 0.1338 | 0.095 | | 1 | | 0.337 |  |
| **Langosta–Yal Ku** | | | | | | | | | | |
| Langosta-Yal Ku | **Average** | **SD** | **Ratio** | **Ava** | **Avb** | | **Cum. sum** | | **p** |  |
| Coral | 0.019475 | 0.009074 | 2.1462 | 0.5794 | 0.5802 | | 0.156 | | 0.1949 |  |
| Foram | 0.016914 | 0.012715 | 1.3303 | 0.35 | 0.291 | | 0.291 | | 0.3985 |  |
| *Homotrema* | 0.015154 | 0.012068 | 1.2557 | 0.4573 | 0.4716 | | 0.412 | | 0.0851 |  |
| Urchin | 0.014553 | 0.009996 | 1.4559 | 0.0859 | 0.0961 | | 0.528 | | 0.0271* |  |
| Coralline algae | 0.01403 | 0.00947 | 1.4815 | 0.1407 | 0.0894 | | 0.641 | | 0.0405* |  |
| *Halimeda* | 0.013678 | 0.010874 | 1.2578 | 0.3307 | 0.3896 | | 0.75 | | 1 |  |
| Intraclast | 0.01287 | 0.009982 | 1.2894 | 0.3551 | 0.385 | | 0.853 | | 0.8984 |  |
| Octocoral | 0.010552 | 0.00833 | 1.2669 | 0.1518 | 0.1016 | | 0.937 | | 0.1974 |  |
| Mollusk | 0.007857 | 0.005656 | 1.3891 | 0.2864 | 0.2898 | | 1 | | 0.961 |  |
| **Langosta–Punta Allen C** | | | | | | | | | | |
|  | **Average** | **SD** | **Ratio** | **Ava** | **Avb** | | **Cum. sum** | | **p** |  |
| *Halimeda* | 0.03213 | 0.015288 | 2.1017 | 0.3307 | 0.5044 | | 0.221 | | 0.1074 |  |
| Coral | 0.0203 | 0.014199 | 1.4299 | 0.5794 | 0.5271 | | 0.361 | | 0.0985 |  |
| Foram | 0.01798 | 0.012746 | 1.4105 | 0.35 | 0.283 | | 0.484 | | 0.2315 |  |
| Intraclast | 0.01548 | 0.011068 | 1.3991 | 0.3551 | 0.4067 | | 0.591 | | 0.5998 |  |
| Coralline algae | 0.01431 | 0.011009 | 1.2999 | 0.4573 | 0.4288 | | 0.69 | | 0.1787 |  |
| Octocoral | 0.01239 | 0.009687 | 1.2794 | 0.1518 | 0.0871 | | 0.775 | | 0.0163* |  |
| Urchin | 0.01162 | 0.008857 | 1.3121 | 0.1407 | 0.1439 | | 0.855 | | 0.3922 |  |
| *Homotrema* | 0.0108 | 0.008726 | 1.2375 | 0.0859 | 0.0867 | | 0.929 | | 0.7068 |  |
| Mollusk | 0.01031 | 0.007473 | 1.3796 | 0.2864 | 0.2389 | | 1 | | 0.3437 |  |
| **Langosta–San Antonio** | | | | | | | | | | |
|  | **Average** | **SD** | **Ratio** | **Ava** | **Avb** | | **Cum. sum** | | **p** |  |
| *Halimeda* | 0.04086 | 0.018059 | 2.2625 | 0.3307 | 0.5522 | | 0.252 | | 0.0003*** |  |
| Foram | 0.02612 | 0.016117 | 1.6207 | 0.35 | 0.2161 | | 0.413 | | 0.0003*** |  |
| Coral | 0.02217 | 0.015665 | 1.415 | 0.5794 | 0.5132 | | 0.55 | | 0.0131* |  |
| Coralline algae | 0.0163 | 0.011979 | 1.3604 | 0.4573 | 0.4252 | | 0.651 | | 0.0263* |  |
| Intraclast | 0.0135 | 0.010492 | 1.2868 | 0.3551 | 0.4026 | | 0.734 | | 0.8496 |  |
| Octocoral | 0.01107 | 0.008016 | 1.3804 | 0.1518 | 0.0953 | | 0.802 | | 0.1111 |  |
| *Homotrema* | 0.01091 | 0.008005 | 1.3624 | 0.0859 | 0.1034 | | 0.87 | | 0.6851 |  |
| Mollusk | 0.01077 | 0.007647 | 1.4081 | 0.2864 | 0.2436 | | 0.936 | | 0.205 |  |
| Urchin | 0.01036 | 0.007724 | 1.3418 | 0.1407 | 0.1442 | | 1 | | 0.6842 |  |
| **Langosta–Mar F5** | | | | | | | | | | |
|  | **Average** | **SD** | **Ratio** | **Ava** | **Avb** | | **Cum. sum** | | **p** |  |
| Intraclast | 0.03818 | 0.014624 | 2.6109 | 0.3551 | 0.5595 | | 0.212 | | 0.0001*** |  |
| *Halimeda* | 0.0297 | 0.012884 | 2.305 | 0.3307 | 0.1728 | | 0.377 | | 0.3232 |  |
| Foram | 0.0272 | 0.016779 | 1.621 | 0.35 | 0.2093 | | 0.529 | | 0.0001*** |  |
| Coral | 0.02228 | 0.014499 | 1.5363 | 0.5794 | 0.6573 | | 0.652 | | 0.0106* |  |
| Coralline algae | 0.01766 | 0.011949 | 1.4782 | 0.4573 | 0.396 | | 0.751 | | 0.0048** |  |
| Urchin | 0.01313 | 0.009987 | 1.3152 | 0.1407 | 0.118 | | 0.824 | | 0.1216 |  |
| *Homotrema* | 0.01215 | 0.009331 | 1.3022 | 0.0859 | 0.111 | | 0.891 | | 0.38 |  |
| Octocoral | 0.01178 | 0.008589 | 1.371 | 0.1518 | 0.095 | | 0.957 | | 0.0445* |  |
| Mollusk | 0.00777 | 0.005547 | 1.4011 | 0.2864 | 0.2785 | | 1 | | 0.9671 |  |
| **Yal Ku–Mar F5** | | | | | | | | | | |
|  | **Average** | **SD** | **Ratio** | **Ava** | **Avb** | | **Cum. sum** | | **p** |  |
| *Halimeda* | 0.04111 | 0.010939 | 3.758 | 0.3896 | 0.1728 | | 0.25 | | 0.0002*** |  |
| Intraclast | 0.03292 | 0.008945 | 3.68 | 0.385 | 0.5595 | | 0.451 | | 0.0001*** |  |
| Foram | 0.01653 | 0.010698 | 1.546 | 0.291 | 0.2093 | | 0.552 | | 0.4616 |  |
| *Homotrema* | 0.01531 | 0.009911 | 1.545 | 0.4716 | 0.396 | | 0.645 | | 0.0796 |  |
| Coralline algae | 0.01511 | 0.008822 | 1.712 | 0.5802 | 0.6573 | | 0.737 | | 0.9175 |  |
| Coral | 0.01472 | 0.010376 | 1.419 | 0.0961 | 0.111 | | 0.827 | | 0.0218* |  |
| Urchin | 0.01109 | 0.008295 | 1.336 | 0.0894 | 0.118 | | 0.894 | | 0.514 |  |
| Octocoral | 0.00895 | 0.007461 | 1.2 | 0.1016 | 0.095 | | 0.949 | | 0.594 |  |
| Mollusk | 0.00836 | 0.005932 | 1.41 | 0.2898 | 0.2785 | | 1 | | 0.9089 |  |
| **Punta Allen N–Mar F5** | | | | | | | | | | |
|  | **Average** | **SD** | **Ratio** | **Ava** | **Avb** | | **Cum. sum** | | **p** |  |
| *Halimeda* | 0.05654 | 0.013926 | 4.06 | 0.4718 | 0.1728 | | 0.339 | | 0.0001*** |  |
| Intraclast | 0.03044 | 0.008007 | 3.802 | 0.3979 | 0.5595 | | 0.521 | | 0.0001*** |  |
| Coral | 0.01808 | 0.011301 | 1.599 | 0.5647 | 0.6573 | | 0.629 | | 0.4498 |  |
| Foram | 0.01326 | 0.009568 | 1.386 | 0.2716 | 0.2093 | | 0.709 | | 0.9115 |  |
| Urchin | 0.01121 | 0.008536 | 1.313 | 0.1104 | 0.118 | | 0.776 | | 0.4907 |  |
| *Homotrema* | 0.00982 | 0.008293 | 1.184 | 0.0912 | 0.111 | | 0.835 | | 0.8806 |  |
| Octocoral | 0.00972 | 0.00746 | 1.303 | 0.1105 | 0.095 | | 0.893 | | 0.3779 |  |
| Mollusk | 0.00903 | 0.006987 | 1.293 | 0.4334 | 0.396 | | 0.947 | | 0.965 |  |
| Coralline algae | 0.00883 | 0.006602 | 1.337 | 0.2534 | 0.2785 | | 1 | | 0.8206 |  |
| **Punta Allen C–Mar F5** | | | | | | | | | | |
|  | **Average** | **SD** | **Ratio** | **Ava** | **Avb** | | **Cum. sum** | | **p** |  |
| *Halimeda* | 0.06273 | 0.013216 | 4.747 | 0.5044 | 0.1728 | | 0.348 | | 0.0001*** |  |
| Intraclast | 0.02872 | 0.010974 | 2.617 | 0.4067 | 0.5595 | | 0.507 | | 0.0001*** |  |
| Coral | 0.02491 | 0.011666 | 2.136 | 0.5271 | 0.6573 | | 0.645 | | 0.0008*** |  |
| Foram | 0.01498 | 0.011232 | 1.334 | 0.283 | 0.2093 | | 0.728 | | 0.7303 |  |
| *Homotrema* | 0.01135 | 0.009254 | 1.226 | 0.0867 | 0.111 | | 0.79 | | 0.5797 |  |
| Urchin | 0.01112 | 0.008835 | 1.258 | 0.1439 | 0.118 | | 0.852 | | 0.5194 |  |
| Mollusk | 0.00955 | 0.007483 | 1.276 | 0.2389 | 0.2785 | | 0.905 | | 0.6076 |  |
| Octocoral | 0.00921 | 0.007759 | 1.187 | 0.0871 | 0.095 | | 0.956 | | 0.5196 |  |
| Coralline algae | 0.00795 | 0.006116 | 1.3 | 0.4288 | 0.396 | | 1 | | 0.9932 |  |
| **San Antonio–Mar F5** | | | | | | | | | | |
|  | **Average** | **SD** | **Ratio** | **Ava** | **Avb** | | **Cum. sum** | | **p** |  |
| *Halimeda* | 0.07172 | 0.016185 | 4.431 | 0.5522 | 0.1728 | | 0.383 | | 0.0001*** |  |
| Intraclast | 0.02963 | 0.00815 | 3.636 | 0.4026 | 0.5595 | | 0.541 | | 0.0001*** |  |
| Coral | 0.02731 | 0.014562 | 1.876 | 0.5132 | 0.6573 | | 0.686 | | 0.0001*** |  |
| *Homotrema* | 0.01025 | 0.0084 | 1.22 | 0.4252 | 0.396 | | 0.741 | | 0.8686 |  |
| Foram | 0.0102 | 0.008007 | 1.274 | 0.2161 | 0.2093 | | 0.796 | | 0.9969 |  |
| Mollusk | 0.01018 | 0.007683 | 1.325 | 0.2436 | 0.2785 | | 0.85 | | 0.3732 |  |
| Urchin | 0.0101 | 0.008451 | 1.195 | 0.1034 | 0.111 | | 0.904 | | 0.8397 |  |
| Coralline algae | 0.00957 | 0.007941 | 1.205 | 0.1442 | 0.118 | | 0.955 | | 0.8397 |  |
| Octocoral | 0.00845 | 0.006783 | 1.245 | 0.0953 | 0.095 | | 1 | | 0.7087 |  |
