# Supplementary material for: Spatial variability of sedimentary assemblages reflects variations in bioerosion pressure of adjacent coral reefs
Source: PLoS One. 2024 Oct 11;19(10):e0311344. doi: 10.1371/journal.pone.0311344 (PMC11469488; doi:10.1371/journal.pone.0311344)
Supplement: S8 Table — Mean (± standard error) relative abundance of sediment grains for all the biological and geological categories found within 12 thin sections of sediments from each site. (DOCX) [file pone.0311344.s014.docx]

**S8 Table. Summary of the composition of sedimentary assemblages across sites.** Mean (± standard error) relative abundance of sediment grains for all the biological and geological categories found within 12 thin sections of sediments from each site.

| **Site** | **Coral** | ***Halimeda*** | **Coralline algae** | **Intraclast** | **Foram** | **Mollusk** | **Urchin** | **Octocoral** | ***Homotrema*** |
| --- | --- | --- | --- | --- | --- | --- | --- | --- | --- |
| Mar F5 | 37.4 ±1.2 | 3.1 ±0.4 | 15.0 ±0.7 | 28.2 ±0.8 | 4.5 ±0.5 | 7.7 ±0.6 | 1.7 ±0.3 | 1.1 ±0.2 | 1.5 ±0.4 |
| Dicks | 32.4 ±2.8 | 11.8 ±1.0 | 19.5 ±1.8 | 12.7 ±1.0 | 11.8 ±1.5 | 5.5 ±0.7 | 2.6 ±0.6 | 1.9 ±0.3 | 1.8 ±0.4 |
| Langosta | 30.5 ±3.1 | 10.8 ±1.1 | 20.0 ±2.1 | 12.5 ±1.5 | 12.3 ±1.7 | 8.1 ±0.6 | 2.4 ±0.5 | 2.4 ±0.3 | 1.0 ±0.3 |
| Yal Ku | 30.1 ±0.8 | 14.5 ±0.9 | 20.8 ±1.2 | 14.2 ±0.8 | 8.4 ±0.8 | 8.3 ±0.6 | 1.0 ±0.2 | 1.2 ±0.2 | 1.5 ±0.4 |
| Punta Allen Centro | 25.4 ±1.3 | 23.5 ±1.3 | 17.3 ±0.6 | 15.8 ±1.1 | 8.0 ±0.9 | 5.7 ±0.5 | 2.3 ±0.4 | 1.0 ±0.2 | 1.0 ±0.3 |
| Punta Allen Norte | 28.8 ±1.4 | 20.9 ±1.4 | 17.7 ±0.8 | 15.1 ±0.7 | 7.3 ±0.7 | 6.4 ±0.6 | 1.5 ±0.3 | 1.4 ±0.3 | 1.0 ±0.2 |
| San Antonio | 24.3 ±1.7 | 27.8 ±2.0 | 17.2 ±1.2 | 15.4 ±0.7 | 4.8 ±0.7 | 6.0 ±0.6 | 2.1 ±0.2 | 1.0 ±0.2 | 1.3 ±0.3 |
