# Supplementary material for: Spatial variability of sedimentary assemblages reflects variations in bioerosion pressure of adjacent coral reefs
Source: PLoS One. 2024 Oct 11;19(10):e0311344. doi: 10.1371/journal.pone.0311344 (PMC11469488; doi:10.1371/journal.pone.0311344)
Supplement: S9 Table — Output from the linear mixed-effect model testing Halimeda grains in the sediment samples as a predictor of live Halimeda cover. Significant predictors are highlighted in gray. (DOCX) [file pone.0311344.s015.docx]

**S9 Table. *Halimeda* sediment grain abundance as a predictor of live *Halimeda* cover.** Output from the linear mixed-effect model testing *Halimeda* grains in the sediment samples as a predictor of live *Halimeda* cover. Significant predictors are highlighted in gray.

|  | **Value** | **SE** | **df** | **t-value** | **p** |
| --- | --- | --- | --- | --- | --- |
| **Intercept** | -1.00 | 0.51 | 34 | -1.96 | 0.06 |
| ***Halimeda* grains** | 0.18 | 0.03 | 34 | 6.40 | <0.0001*** |
