# Supplementary material for: Spatial variability of sedimentary assemblages reflects variations in bioerosion pressure of adjacent coral reefs
Source: PLoS One. 2024 Oct 11;19(10):e0311344. doi: 10.1371/journal.pone.0311344 (PMC11469488; doi:10.1371/journal.pone.0311344)
Supplement: S10 Table — Tukey HSD post-hoc, pairwise comparisons of the mean abundance of coral grains of all localities. Significant comparisons are highlighted in gray. (DOCX) [file pone.0311344.s016.docx]

**S10 Table. Post-hoc, pairwise comparison of sedimentary assemblages across localities.** Tukey HSD post-hoc, pairwise comparisons of the mean abundance of coral grains of all localities. Significant comparisons are highlighted in gray.

| **Pairs** | **Difference** | **Lower Interval** | **Upper Interval** | **p** |
| --- | --- | --- | --- | --- |
| Punta Allen-Akumal | -4.811111 | -7.481089 | -2.141133 | 0.000 *** |
| Punta Maroma-Akumal | 6.394444 | 2.618525 | 10.170364 | 0.001** |
| Punta Maroma-Punta Allen | 11.205556 | 7.429636 | 14.981475 | 0.000*** |
